# Highly bent crystals formed by restrained π-stacked columns connected *via* alkylene linkers with variable conformations†

**DOI:** 10.1039/c4sc03849e

**Published:** 2015-02-03

**Authors:** Chih-Ming Chou, Shunpei Nobusue, Shohei Saito, Daishi Inoue, Daisuke Hashizume, Shigehiro Yamaguchi

**Affiliations:** a Department of Chemistry, Graduate School of Science, and Institute of Transformative Bio-Molecules (WPI-ITbM), Nagoya University Furo, Chikusa Nagoya 464-8602 Japan yamaguchi@chem.nagoya-u.ac.jp +81 52-789-5947; b Materials Characterization Support Unit, RIKEN Center for Emergent Matter Science (CEMS) 2-1 Hirosawa Wako Saitama 351-0198 Japan

## Abstract

A reproducible formation of strongly bent crystals was accomplished by structurally restraining macrocyclic π-conjugated molecules. The model π-units consist of two 9,10-bis(2-thienylethynyl)anthracenes with a strong propensity for stacking, which are connected in a macrocyclic fashion *via* two alkylene linkers. The correlation between the crystalline morphology and the macrocyclic structures restrained by a variety of flexible alkylene linker combinations was systematically studied. Bent crystals were obtained only with specific alkylene linkers of appropriate chain length. The alkylene linkers can adopt different conformations in the crystal packing, so as to fill voids within the macrocycle. The ability to form several similar molecular structures with different alkylene conformations gives rise to contaminations of different crystalline phases within a single crystal, and it is these phase contaminations which are responsible for the bending of the crystals.

## Abstract 2

Highly bent organic crystals are reproducibly prepared using a structurally restrained macrocyclic π-conjugated system with two flexible linkers. The appropriate length of the linkers to produce a void within the macrocycle is key to the observed bending of the crystals.

## Graphical abstract

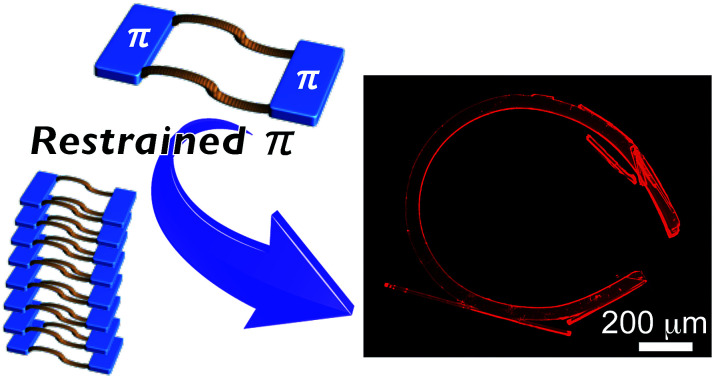

## Introduction

The properties of π-conjugated molecules in the crystalline state highly rely on the spatial arrangement of the molecules in the crystal structure. The control over the molecular order, and consequently the crystal growth (crystal engineering)^1^ enables the induction of unusual properties, such as charge carrier transport,^2^ mechanochromic luminescence,^3^ optical waveguide with laser oscillation,^4^ and optical nonlinearity.^5^ In this context, organic crystals that can undergo morphological changes upon external stimuli, including mechanical stress,^6^ light,^7^ and heat,^8^ have attracted increasing attention. Desiraju and Reddy have demonstrated that mechanical bending of organic crystals can be achieved by a non-covalent approach, in which the crystal structures are constructed anisotropically, based on strong hydrogen bonds and weak intermolecular interactions in orthogonal directions to each other.^6*a*–*c*^ Photoresponsive organic crystals exhibiting bending or twisting behavior have also been reported for several photoactive molecules, including diarylethene, azobenzene, and anthracene derivatives.^7^ Their dynamic behavior is generally based on a morphological change on the surface of single crystals. On the other hand, the thermal response of organic crystals with a variety of dynamic motions is also of great interest.^8^ Thermal phase transitions eventually proceed through the whole crystal, which results in a straightening of the crystals *via* further heating processes following the bending motion.^8*a*^

**Fig. 1:**
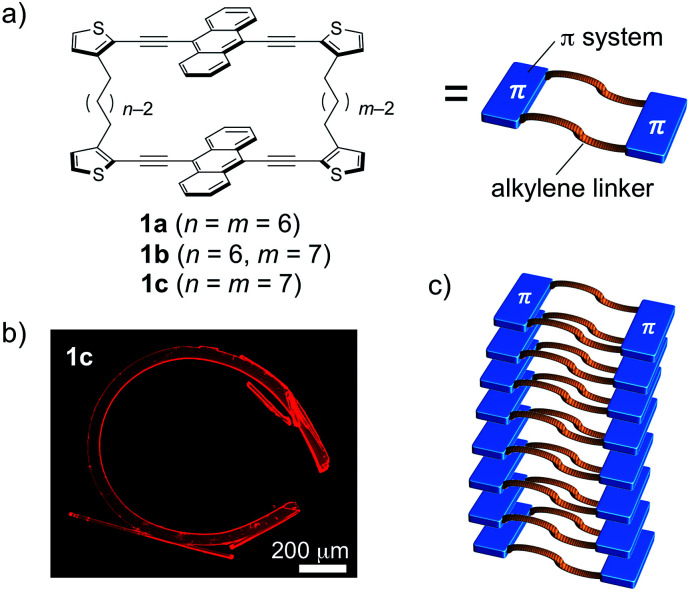
(a) Macrocyclic dimers of 9,10-bis(2-thienylethynyl)anthracene 1a–c connected *via* various combinations of alkylene linkers, (b) a bent crystal of 1c·bent, and (c) schematic representation of the packing structure that leads to the bent crystal shape on a macroscopic scale.

Recently, we proposed the crystal-engineering concept of “macrocyclic restriction”,^9^ where the molecular arrangement in the crystalline state can be controlled by a restriction of the orientation of the π-conjugated skeletons, which are covalently bound to flexible linkers forming a macrocycle. On the basis of this concept, distinct packing structures of cyclic terthiophene dimers with a variety of alkylene linkers were produced. The degree of intermolecular van der Waals interactions was altered by different conformations of the macrocyclic molecules. As a result, their fluorescence and gelation properties were largely perturbed by changing the combination of the flexible alkylene linkers.^9^ We envisioned that this concept should also be applicable to various other π-conjugated skeletons. Following this notion, we selected 9,10-bis(2-thienylethynyl)anthracene, as it represents a more extended π-skeleton, which has a strong propensity towards π-stacking and an intense fluorescence (Fig. 1).^10^ During the course of a systematic study on a series of the macrocyclic dimers 1a–c containing different combinations of alkylene linkers, we discovered that 1c can reproducibly form highly bent crystals. Notably, the bent shape was formed during the crystal growth under thermal conditions in the absence of any other external stimuli.^11^ In this article, we report details of this intriguing macroscopic bending and of its origin.

## Results and discussion

Compounds 1a–c consist of two 9,10-bis(2-thienylethynyl)anthracenes, which are connected at the 3-positions of the terminal thiophene rings *via* different combinations of two alkylene linkers (hexylene or heptylene; Fig. 1a). Detailed synthetic procedures are described in the ESI (Scheme S1).† Single crystals of 1a–c were obtained by vapor diffusion of 2-propanol into 1,2-dichloroethane (DCE) solutions of these compounds, and crystallographic details are listed in Table 1.

**Table 1:** Crystallographic data for the crystals of 1a–c

	1a	1b	1c·bent HT^a^	1c·bent LT^a^
Formula	C_64_H_48_S_4_	C_65_H_50_S_4_	C_66_H_52_S_4_	C_66_H_52_S_4_
*T* (°C)	−150	−150	20	−150
Space group	Monoclinic *P*2_1_/*n*	Monoclinic *P*2_1_/*n*	Monoclinic *P*2_1_/*n*	Monoclinic *P*2_1_/*n*
*a* (Å)	20.159(2)	20.100(2)	21.430(5)	20.0834(7)
*b* (Å)	5.0166(5)	5.1652(4)	5.2798(12)	5.2324(2)
*c* (Å)	25.728(3)	25.159(3)	24.950(6)	24.6005(8)
*β* (°)	109.164(4)	109.501(6)	111.599(5)	109.019(2)
*V* (Å^3^)	2457.7(5)	2462.2(4)	2624.8(11)	2444.01(15)
*Z*	2	2	2	2
GOF	1.043	1.104	0.887	1.036
*R*_int_	0.0633	0.0164	0.0824	0.0707
*R*(*F*)	0.0572	0.0415	0.0778	0.0635
w*R*(*F*^2^)	0.1318	0.1060	0.2686	0.1873
CCDC	1013992	1013993	1013995	1013996

a The crystal was annealed at 100 °C for 30 min and then slowly cooled down to the corresponding temperature (Δ*T*/Δ*t* = 1 °C min^−1^).

**Fig. 2:**
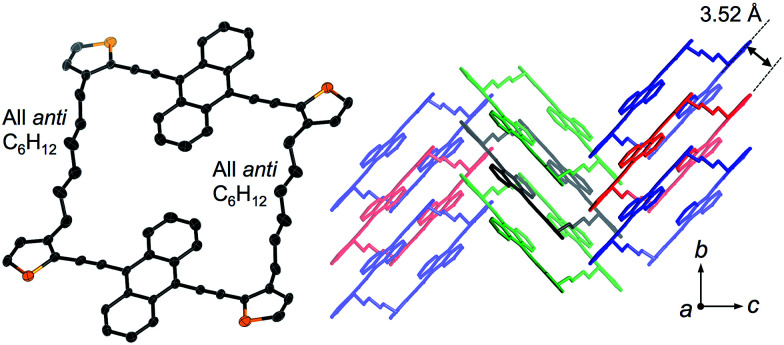
X-ray crystal structure of macrocyclic dimer 1a, showing the molecular structure (left) and the crystal packing (right). Thermal ellipsoids are drawn at a probability level of 50%.

Crystals of 1a and 1b belong to the monoclinic *P*2_1_/*n* space group, and exhibited a comparable herringbone packing structure, irrespective of the alkylene chain length (Fig. 2 and S1†). In these structures, the bis(thienylethynyl)anthracenes retain a highly planar conformation. In each molecule, the two π-conjugated moieties are aligned in parallel, even though no intramolecular overlap between these π-skeletons was observed. For 1a and 1b, the intramolecular distances between the centroids of the bis(thienylethynyl)anthracenes were measured to be 10.73 Å and 10.77 Å, respectively. Along the *b*-axis, the packing structures exhibited a slipped face-to-face alignment of the π-conjugated skeletons, *i.e.* an anthracene moiety in one molecule is positioned above an acetylene moiety in one of the adjoining molecules. The mean-plane distances between two adjacent π-skeletons were observed to be 3.52 Å (1a) and 3.53 Å (1b). Between stacked columns, neighboring π-skeletons adopt an edge-to-face arrangement.

**Fig. 3:**
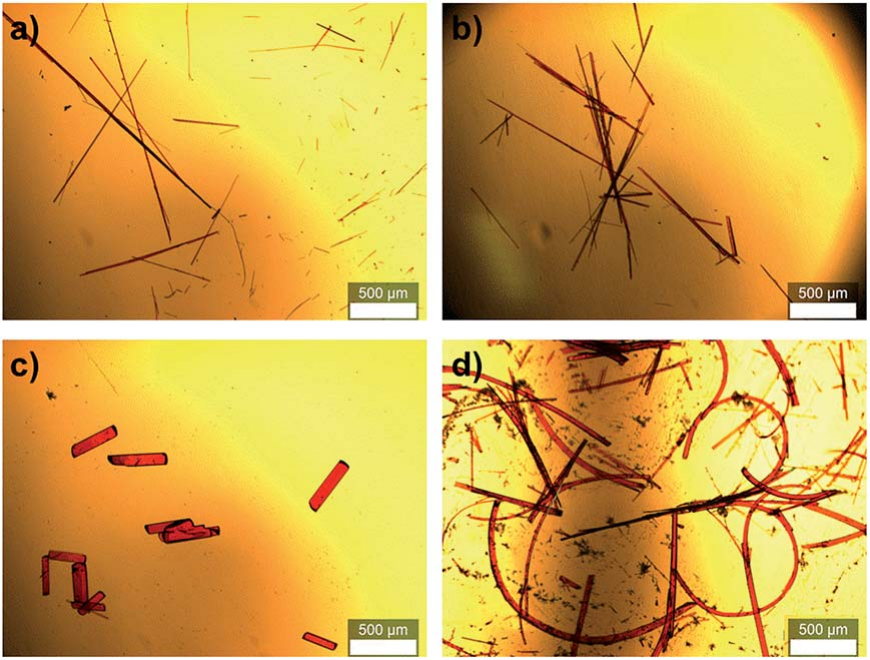
Optical micrographs of macrocyclic dimers: (a) 1a, (b) 1b, (c) 1c·prism, cocrystallized with 1,2-dichloroethane, and (d) highly curved 1c·bent. The scale bar represents 500 μm.

An important feature in the structures of 1a and 1b is that the alkylene linkers are partially flexible, and can thus modify their conformations in order to preserve the parallel orientation of the two π-skeletons. For example, compound 1a contains two stretched hexylene linkers and adopted an all-*anti* conformation (Fig. 2). Even though compound 1b also exhibited a stretched all-*anti* conformation for the hexylene linker, it showed a distorted structure with three *gauche* moieties for the heptylene linker (Fig. S1†). The disorder of these linkers shares the same ratio of occupancy, due to the crystallographic inversion symmetry at the center of the macrocycle. As evident from the optical micrographs, crystals of 1a and 1b exhibited a needle crystal habit (Fig. 3a and b). The determination of the face indexes for 1a and 1b suggested that the longitudinal direction of the needle crystals corresponds to the direction of the molecular stacking along the *b* axis (Fig. S2†), which is consistent with calculations on the crystal morphology using the Bravais–Friedel–Donnay–Harker (BFDH) method (Fig. S3†).^12^ Moreover, these crystals showed elastic bending behavior in response to mechanical force (Fig. S4†).^6^

In contrast to 1a and 1b, the macrocyclic dimer 1c, bearing two heptylene linkers, formed two kinds of crystals (1c·prism and 1c·bent). These crystals were obtained from the same solvent system, albeit at different temperatures and concentrations. Single crystals of 1c·prism were obtained in a week by vapor diffusion of 2-propanol into a dilute DCE solution of 1c (0.5 mg of 1c in 2 mL of DCE, 0.25 mM) at room temperature (20 °C). These crystals were rectangular in habit, and contained molecules of DCE as crystal solvent (Fig. 3c, S5 and Table S1†). In this crystal structure, both heptylene linkers adopt a partially stretched conformation with one *gauche* unit.

**Fig. 4:**
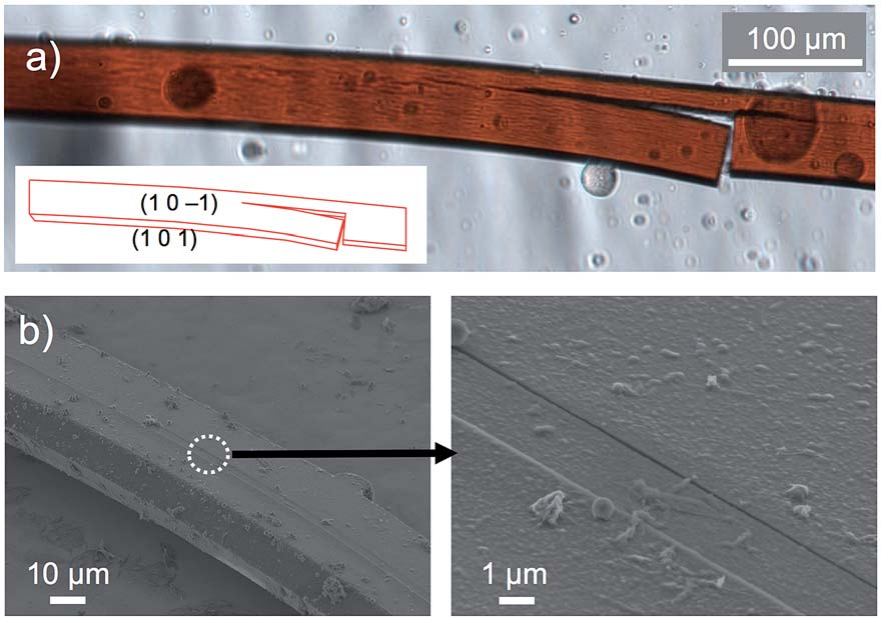
(a) Optical micrograph of cracked bent crystals of 1c·bent (inset: face index assignment) and (b) SEM images of 1c·bent.

On the other hand, crystals of 1c·bent were obtained from a hot, saturated solution of 1c in DCE at 70 °C (4.4–5.8 mg of 1c in 4 mL of DCE; 2.2–3.0 mM). The hot solution was exposed to a vapour of 2-propanol before the temperature went down. Then the 1c·bent crystals were formed at ambient temperature within less than half a day, whereas leaving the solution in the absence of 2-propanol only resulted in the rapid precipitation of a powder sample from the supersaturated state. Much to our surprise, the optical micrographs of these crystals showed significantly bent shapes, with arc angles of up to 300° (Fig. 1b and 3d). Notably, recrystallization under identical conditions afforded these bent crystals reproducibly. In contrast to the discontinuous microcrystal assemblies discussed in some previous reports,^13^ scanning electron microscopic (SEM) images of 1c·bent revealed a highly continuous surface (Fig. 4b and S6†). Only minor cracks (micro- to millimeter-scale) were observed in the longitudinal direction, which are most likely formed to partially release the bending strain.

Unfortunately, all attempts to conduct single crystal X-ray diffraction analyses on these bent crystals were unsuccessful. The observed reflection spots were thin and positively biased in the circumferential direction, indicative of the presence of a crystalline phase in combination with a less ordered packing structure (Fig. S7†).^6*c*^ Even when the bent crystal 1c·bent was cut into small pieces for the structural determination in the local area of the crystal, the X-ray diffraction was not improved presumably due to the large conformational heterogeneity. However, brief annealing of the small crystal piece (*T* = 100 °C, *t* = 30 min) changed the elliptic diffraction spots and significantly improved the data quality, thus producing a more uniform atomic displacement and permitting the analysis of the X-ray crystal structure. In conjunction with this change, we also observed that the macroscopic shape of the crystal changed during the annealing process from bent to straight (Fig. S8†), even though the dynamic motion was slow (*ca.* 40 μm h^−1^) and only observed for relatively thin crystals.

**Fig. 5:**
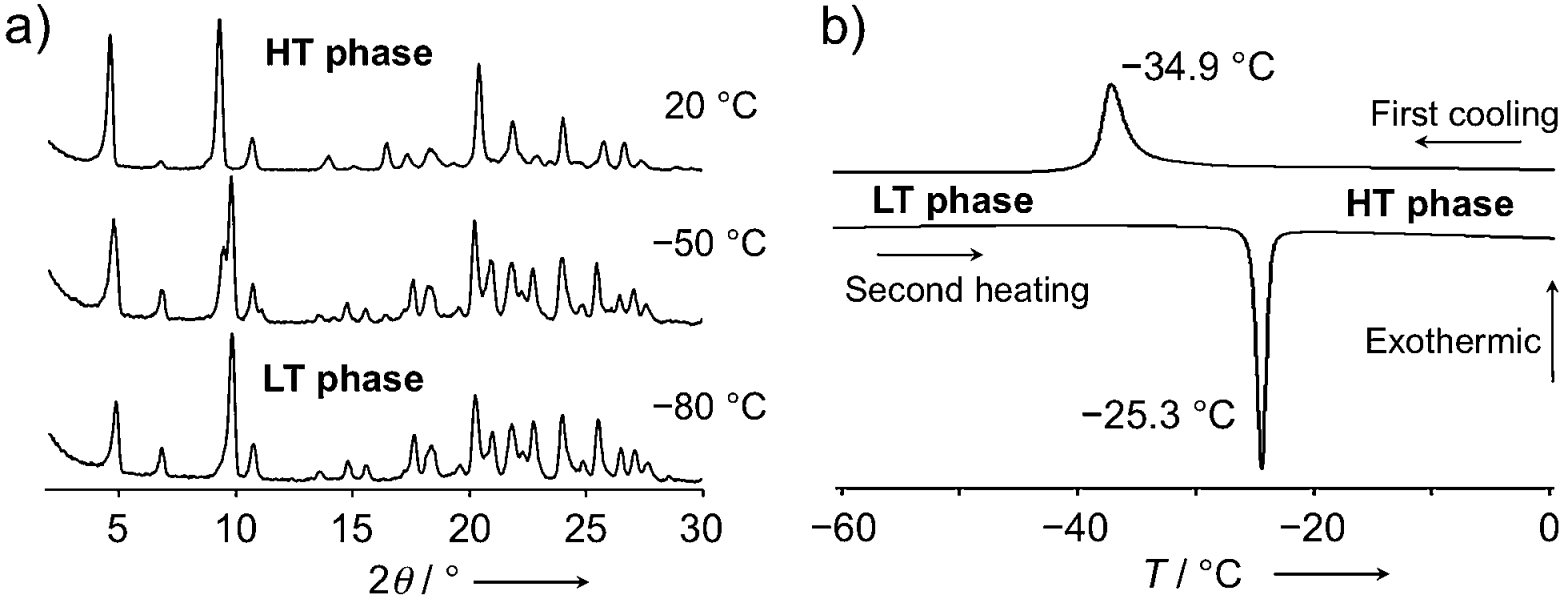
(a) Powder X-ray diffraction patterns of crystalline 1c·bent at 20, −50, and −80 °C. (b) DSC profile of a 1c·bent crystal (Δ*T*/Δ*t* = 2 °C min^−1^).

To gain deeper insight into the thermal behavior, variable-temperature powder X-ray diffraction patterns were measured between 100 °C and −80 °C (10 °C intervals). Whereas the packing structure was not significantly affected by temperature, a reversible thermal phase transition was observed between −20 °C and −60 °C (Fig. 5a, S9 and S10†). Differential scanning calorimetry (DSC) measurements of 1c·bent (Δ*T*/Δ*t* = 2.0 °C min^−1^) reproducibly generated an exothermic peak at −34.9 °C (Δ*H* = 6.40 kJ mol^−1^) during the cooling process, as well as an endothermic peak at −25.3 °C (Δ*H* = 6.55 kJ mol^−1^) during the subsequent second heating process (Fig. 5b). This phase transition behavior, as well as the degree of the enthalpy change, are typical for conformational changes in flexible chains.^8*a*^ In contrast, 1a and 1b did not show any such phase transition in comparable DSC measurements (Fig. S11†), demonstrating that the phase transition observed for the crystals of 1c·bent is a unique feature of their bent shape. In the following discussion, phases in higher or lower temperature regions relative to the transition point will be referred to as high temperature (HT) or low temperature (LT) phase, respectively.

**Fig. 6:**
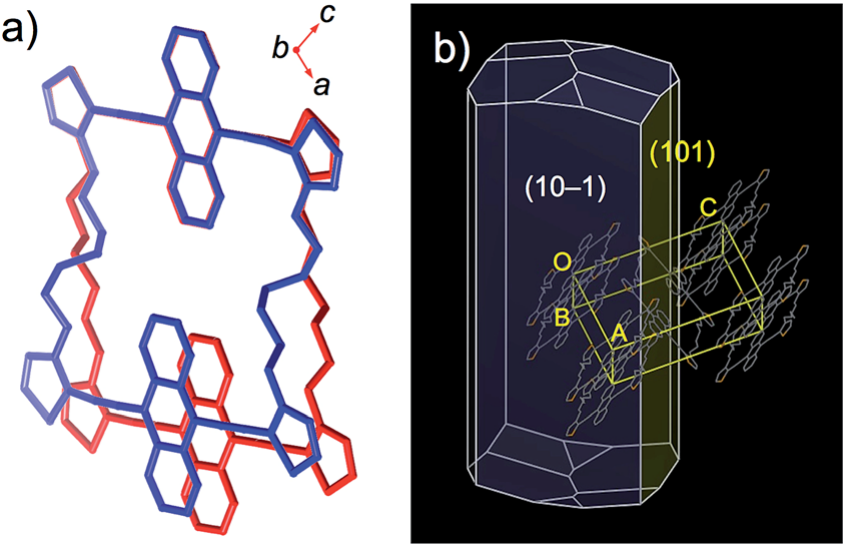
(a) Superimposed crystal structures of 1c·bent at 20 °C (HT; red) and −150 °C (LT; blue). The inserted axes correspond to the geometry at 20 °C. (b) BFDH morphology calculation of the 1c·bent crystal structure at 20 °C.

After many trials, we succeeded in obtaining crystal structures for 1c·bent by conducting a careful pretreatment of the examined crystals. For the pretreatment, an apparently straight part of the 1c·bent crystal was cut out into small pieces and annealed (*T* = 100 °C, *t* = 30 min), followed by slow cooling (Δ*T*/Δ*t* = 1 °C min^−1^). Using the crystals thus obtained, we were able to determine two different crystal structures at 20 °C and −150 °C, which correspond to the HT and LT phases, respectively (Fig. 6). These crystal structures exhibit similar unit cell parameters, and their space group (*P*2_1_/*n*) is the same as in 1a and 1b (Table 1). However, a comparison between the structures of the HT and LT phase revealed different conformations of the alkylene moieties, which corroborates the DSC analysis (Fig. 6a). In the HT phase at 20 °C, the heptylene linkers are stretched, adopting a conformation with one *gauche* and one *eclipsed* unit, resulting in a relatively flat macrocyclic structure. The thermal ellipsoids of the inner carbon atoms in the heptylene chains are significantly larger than those in the rigid aromatic units (Fig. S12†). In contrast, the thermal ellipsoids at −150 °C were well converged, even those in the heptylene linkers (Fig. S13†). In the LT phase, the heptylene linkers maintain a more distorted conformation with three *gauche* units, so as to fill voids within the macrocycle. Upon changing from HT to LT phase, the distance between the centroids of the π-skeletons decreased from 11.58 Å to 10.71 Å, while the intramolecular distance between the mean planes of the two bis(thienylethynyl)anthracene moieties increased from 1.57 Å to 2.68 Å.

These results suggest that the large void enclosed by the two heptylene-linked macrocycles in 1c is responsible for its unique thermal phase transition behavior. In the HT phase, the void in 1c is occupied by the large thermal displacement of the atoms in the flexible heptylene chains. However, this thermal vibration is suppressed at lower temperature (LT phase), and thus the heptylene chains change their conformations to fill the void. As evident from their space-filling plots (Fig. S14†), compounds 1a (hexylene–hexylene) and 1b (hexylene–heptylene) contain, due to the shorter linkers, smaller voids compared to 1c. Therefore, these compounds do not show the thermal phase transition behavior observed for 1c.

By analysis of the optical microscopy images and BFDH morphology calculations, the bending direction in 1c·bent was determined to be perpendicular to the (101) plane, which is parallel to the *b* axis (Fig. 4a and 6b). This result indicates that while the columnar π-stacked structure is essentially preserved along the *b* axis, the columns are gradually bent as the molecules slide in the lateral direction of the dithienylalkane moiety.^6^ The comparison of the unit cell parameters showed that the unit cell in the LT phase is anisotropically shrunk relative to that in the HT phase (Table S2†). Thus, upon changing from the HT phase at −20 °C (*a* = 21.60 Å, *b* = 5.31 Å, *c* = 24.31 Å, *β* = 111.9°) to LT phase at −60 °C (*a* = 20.21 Å, *b* = 5.27 Å, *c* = 24.70 Å, *β* = 109.1°), the *a* axis is significantly contracted by −6.4% and the *β* angle becomes narrower by −2.8°, whereas the *b* and *c* axes are only changed by −0.7% and +1.6%, respectively. These results suggest that the formation of the bent crystals is most likely due to a contamination of the anisotropically shrunk LT phase in the dominant HT phase. To support this assumption, we measured a powder X-ray diffraction pattern of the freshly prepared 1c·bent crystal before annealing. By means of the Pawley method,^14^ the observed pattern was successfully fitted as a mixture of the HT and LT phases (Fig. S15 and Table S3†). These results offered the contamination of both these phases. In consideration of the anisotropically curved shape of the 1c·bent crystals, the contamination of the LT phase should occur in an inhomogeneous fashion, as a homogeneous distribution would lead to a straight-shaped crystal especially with the crystallographic inversion symmetry at the center of the macrocycle.

In light of the previously discussed results, we propose the following mechanism for the crystal growth and thermal transformation of the bent crystal. When crystals of 1c are prepared from a hot saturated DCE solution, the crystallites in the HT phase are rapidly stacked by van der Waals interactions. However, trace amounts of LT phase crystallites are probably included in an inhomogeneous fashion, as it is an energetically close form, resulting in the bending of the crystal. Annealing of the crystal induces a phase transition in the trace amounts of LT phase crystallites, rendering the crystal more uniformly HT phase. This phase transition is responsible for the observed slow straightening motion of the thin crystals. Varying the contamination ratio between the LT and HT phase may lead to different degrees of curvature in the 1c·bent crystals. The origin of the inhomogeneous distribution of the LT phase in the parent HT phase remains unclear so far, although local temperature gradients in the hot solution might be responsible.^11^

The fluorescence spectra in the crystalline state also provided some structural information about these crystals (Fig. S17†). Firstly, broad fluorescence bands (*λ*_max_ = 620 nm) were observed for crystals of both 1a and 1b. The similarity of their spectra is consistent with the similar arrangement of the π-skeletons in their packing structures. Secondly, only the fluorescence spectrum of 1c·prism crystals showed two bands at 582 and 616 nm, which are hypsochromically shifted compared to those of 1a and 1b. This feature should arise from different packing structures due to the inclusion of solvent molecules. Thirdly, compared with the crystals of 1a and 1b, a broad and bathochromically shifted emission band (*λ*_max_ = 631 nm) was observed in the bent crystal of 1c·bent. Annealing of this crystal resulted in a slight hypsochromic shift (*λ*_max_ = 625 nm), under concomitant sharpening of the emission band. The spectral sharpening is thereby indicative of a more uniform arrangement of the π-conjugated skeleton posterior to annealing.

## Conclusions

We have disclosed the unusual formation of highly bent organic crystals of structurally restrained macrocyclic dimers of 9,10-bis(2-thienylethynyl)anthracene linked *via* two alkylene chains. The selection of alkylene linkers of appropriate length is crucial for the formation of bent crystals, as it largely influences the morphology of the crystals. Long alkylene chains in the macrocyclic structure adopt various conformations by including *gauche* or *eclipsed* units into the *anti*-conformation chain, so as to fill the large void within the macrocycle in the crystal packing. As a consequence, the macrocycle 1c, containing long heptylene linkers, produced two different crystal structures, the formation of which is the origin of the highly bent crystals. To the best of our knowledge, crystal bending of π-conjugated molecules simply by tuning the lengths of covalent alkylene linkers is unprecedented. This work should provide an intriguing design principle for π-conjugated molecules with a specific crystal shape and potential applications in optical materials or optoelectronic micro/nano devices.

## Supplementary Material

SC-006-C4SC03849E-s001

SC-006-C4SC03849E-s002
