# Supplementary material for: Highly bent crystals formed by restrained π-stacked columns connected via alkylene linkers with variable conformations
Source: Chem Sci. 2015 Feb 3;6(4):2354–9. doi: 10.1039/c4sc03849e (PMC5645777; doi:10.1039/c4sc03849e)
Supplement: SC-006-C4SC03849E-s001 [file SC-006-C4SC03849E-s001.pdf]

Electronic Supplementary Material (ESI) for Chemical Science.
This journal is © The Royal Society of Chemistry 2015

Highly Bent Crystals Formed by Restrained π-Stacked Columns Connected *via* Alkylene Linkers with Variable Conformations

Chih-Ming Chou, Shunpei Nobusue, Shohei Saito,* Daishi Inoue, Daisuke Hashizume and Shigehiro Yamaguchi*

Department of Chemistry, Graduate School of Science, and Institute of Transformative Bio-Molecules (WPI-ITbM), Nagoya University, Furo, Chikusa, Nagoya 464-8602, Japan.

Materials Characterization Support Unit, RIKEN Center for Emergent Matter Science (CEMS), 2-1 Hirosawa, Wako, Saitama 351-0198, Japan

SUPPLEMENTARY INFORMATION

Table of Contents

I.	Experimental details	S3
II.	Crystallographic data, mechanical bending behavior, SEM images, powder X-ray diffraction patterns, and DSC profiles	S10
III.	Fluorescence spectra of the crystals	S20
IV.	^{1}H and ^{13}C NMR spectra	S21

I. Experimental details

General. ^{1}H and ^{13}C NMR spectra were recorded with a JEOL AL-400 MHz spectrometer (400 MHz for ^{1}H and 100 MHz for ^{13}C). Chemical shifts are reported in δ ppm using $CHCl_3$ (7.26 ppm) for ^{1}H NMR, and $CDCl_3$ (77.16 ppm) for ^{13}C NMR as an internal standard. Mass spectrometry was measured with a Bruker Daltonics MicroTOF focus using a positive-mode APCI-TOF method in a toluene solution and a Bruker Daltonics Ultraflex III TOF/TOF (MALDI-TOF-MS). Melting points (Mp.) were measured on a Yanaco MP-S3 instrument. Single crystal X-ray diffraction measurements were performed with Rigaku X-ray diffractometers. For the **1a** and **1c·prism** crystals, the diffractometer equipped with a molybdenum MicroMax-007 generator, VariMax-Mo optics, and Saturn70 CCD detector was used. For the **1b** crystal, the diffractometer equipped with a molybdenum FR-X generator, VariMax-Mo optics, and a PILATUS 200K detector was used. For **1c·bent**, the diffractometer equipped with a copper MicroMax-007 microfocus generator, VariMax-Cu optics, and a RAPID IP detector was used. Temperature varied powder X-ray diffraction patterns were measured with a Rigaku SmartLab X-ray diffractometer, equipped with AnthonPaar DCS 350 temperature controller, using Cu $K\alpha$ radiation in 2θ / θ mode at the rate of 2 ° min^{-1}. Temperature was varied at 2 °C min^{-1}, and annealed 5 min before diffraction measurements at each temperature. The pattern resolution and unit-cell refinement of powder X-ray diffraction was performed by Pawley method with Rigaku PDXL2. For scanning electron microscopy, the crystals of **1** in 1,2-dichloroethane solution were dried on a silicon-wafer, and were coated with osmium using Filgen, OPC80N osmium coater. Secondary electron images were observed using a JEOL JSM-6330F scanning electron microscope at accelerating voltage of 3 kV with 45° tilting of the sample stage. Fluorescence spectra of the crystals were measured with a Hitachi F-4500 spectrometer in spectral grade solvents. Column chromatography was performed using neutral silica gel PSQ 60B or PSQ100B (Fuji Silysia Chemical). All reactions were performed under a nitrogen atmosphere, unless stated otherwise. Commercially available solvents and reagents were used without further

purification unless otherwise mentioned. Dry ether, toluene, tetrahydrofuran (THF) and diisopropylamine were purchased from Kanto Chemical. Compounds **2a**, **2b,** and 9-bromo-10-iodoanthracene were prepared according to the literature methods.[1,2]

[1] S. Saito, K. Nakakura and S. Yamaguchi, *Angew. Chem. Int. Ed.,* 2012, **51**, 714.
[2] E. E. Nesterov, Z. Zhu and T. M. Swager, *J. Am. Chem. Soc.*, 2005, **127**, 10083.

Scheme S1

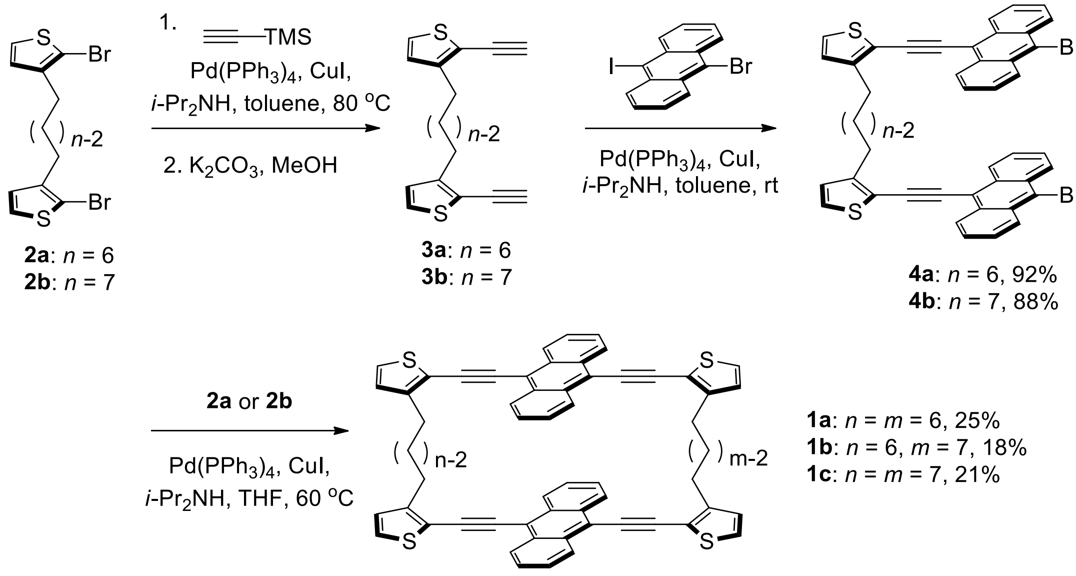

A general procedure for the Sonogashira coupling/desilylation reaction (I).

To a 100 mL two-necked flask 1,6-bis(2-bromo-3-thienyl)hexane or 1,7-bis(2-bromo-3-thienyl)-heptane (1.0 equiv.), trimethylsilylacetylene (2.3 equiv.), $Pd(PPh_3)_4$ (5.0 mol%) and CuI (5.0 mol%) in dry toluene/diisopropylamine (3:1) were added. The resulting mixture was stirred at 80 °C under a nitrogen atmosphere for 12 h. The resulting mixture was extracted with CH_2Cl_2, washed with water and dried over anhydrous $MgSO_4$. After the solvent was removed under reduced pressure, the reaction mixture was treated with K_2CO_3 (3.0 equiv.) in MeOH/THF and stirred at room temperature. After the reaction completed, water was added and the mixture was extracted with ether. The combined organic layer was washed with water and brine dried over anhydrous $MgSO_4$, filtered, and concentrated under reduced pressure. Purification by flash column chromatography on silica gel afforded the corresponding terminal alkyne products.

A general procedure for the Sonogashira coupling reaction (II).

To a 100 mL two-necked flask 1,6-bis(2-ethynyl-3-thienyl)hexane or 1,7-bis(2-ethynyl-3-thienyl)heptane (1.0 equiv.), 9-bromo-10-iodoanthracene (2.0 equiv.), $Pd(PPh_3)_4$ (5.0 mol%) and CuI (5.0 mol%) in dry toluene/diisopropylamine were added and stirred at room temperature under a

nitrogen atmosphere for overnight. After addition of water, the reaction mixture was extracted with $CHCl_3$. The combined organic layer was washed with water and brine, dried over anhydrous $MgSO_4$, filtered, and concentrated under reduced pressure to afford the desired coupling product and was used without further purification.

A general procedure for the Sonogashira coupling reaction (III).

To a 200 mL three-necked flask the 1,6-bis(2-ethynyl-3-thienyl)hexane or 1,7-bis(2-ethynyl-3-thienyl)heptane (1.0 equiv.), $Pd(PPh_3)_4$ (5.0 mol%) and CuI (5.0 mol%) was placed. Aryl bromide (1.0 equiv.) in dry diisopropylamine/THF was slowly added to the reaction mixture at room temperature and then heated at 60 °C with stirring under a nitrogen atmosphere for 18 h. After removal of the solvents under reduced pressure, water was added and the mixurre was extracted with $CHCl_3$. The combined organic layer was water and brine, dried over anhydrous $MgSO_4$, filtered, and concentrated under reduced pressure. Purification by flash column chromatography on silica gel afforded the desired coupling product.

1,6-Bis(2-ethynyl-3-thienyl)hexane (3a): According to the general procedure **I**: Step 1: **2a** (8.30 g, 20.3 mmol), trimethylsilylacetylene (8.5 mL, 60 mmol), $Pd(PPh_3)_4$ (1.15 g, 1.00 mmol) and CuI (190 mg, 1.00 mmol) in diisopropylamine (20 mL) and toluene (60 mL). Step 2: K_2CO_3 (8.28 g, 59.9 mmol) in MeOH (100 mL) and THF (20 mL). Purification by flash column chromatography (CH_2Cl_2/hexane = 1:9, R_f = 0.50) gave **3a** (4.06 g, 13.6 mmol) in 65% as a yellow liquid: ^{1}H NMR (400 MHz, $CDCl_3$): δ 7.15 (d, J= 5.2 Hz, 2H), 7.84 (d, J= 5.2 Hz, 2H), 3.42 (s, 2H), 2.70 (t, J= 7.6 Hz), 1.50-1.70 (m, 4H), 1.30-1.45 (m, 4H); ^{13}C NMR (100 MHz, $CDCl_3$): δ 148.9, 128.0, 126.2, 117.1, 83.2, 76.8, 30.1, 29.3, 28.9; HRMS (APCI, positive) calculated for $C_{18}H_{18}S_2$ ($[M]^+$): 299.0923. Found: 299.0935.

1,7-Bis(2-ethynyl-3-thienyl)heptane (3b): According to the general procedure **I**: Step 1: **2b** (1.09 g,

2.58 mmol), trimethylsilylacetylene (0.85 mL, 6.0 mmol), $Pd(PPh_3)_4$ (120 mg, 0.104 mmol) and CuI (19.7 mg, 0.103 mmol) in diisopropylamine (4 mL) and toluene (12 mL). Step 2: K_2CO_3 (1.09 g, 7.89 mmol) in MeOH (10 mL) and THF (2 mL). Purification by flash column chromatography (CH_2Cl_2/hexane = 1:9, R_f = 0.50) gave **3b** (475 mg, 1.52 mmol) in 58% as a yellow liquid: ^1H NMR (400 MHz, $CDCl_3$): δ 7.15 (d, J= 5.2 Hz, 2H), 6.84 (d, J= 5.2 Hz, 2H), 3.43 (s, 2H), 2.71 (t, J= 7.8 Hz, 4H), 1.55-1.68 (m, 4H), 1.30-1.40 (m, 6H); ^{13}C NMR (100 MHz, $CDCl_3$): δ 149.0, 128.0, 126.2, 116.97, 83.2, 76.7, 30.1, 29.3, 29.06, 29.05; HRMS (APCI, positive) calculated for $C_{19}H_{20}S_2$ ($[M]^+$): 312.1001. Found: 312.1006.

1,6-Bis[2-(10-bromo-9-anthryl)ethynyl)-3-thienyl]hexane (4a): According to the general procedure **II**: **3a** (624 mg, 2.09 mmol), 9-bromo-10-iodoanthracene (1.60 g, 4.18 mmol), $Pd(PPh_3)_4$ (116 mg, 0.100 mmol) and CuI (19 mg, 0.10 mmol) in diisopropylamine (10 mL) and toluene (10 mL) to give **4a** (1.47 g, 1.82 mmol) in 91% as a yellow solid: Mp.: 165–166 °C; ^1H NMR (400 MHz, $CDCl_3$): δ 8.43–8.55 (m, 8H), 7.50–7.55 (m, 12H), 7.25 (d, J= 5.2 Hz, 2H), 6.89 (d, J= 5.2 Hz, 2H), 2.92 (t, J= 7.8 Hz, 4H), 1.78–1.85 (m, 4H), 1.53–1.60 (m, 4H); ^{13}C NMR (100 MHz, $CDCl_3$): δ 147.9, 132.5, 130.2, 128.5, 127.3, 127.0, 126.73, 126.70, 123.98, 118.5, 118.2, 94.7, 92.1, 30.5, 30.0, 29.3; HRMS (APCI, positive) calculated for $C_{46}H_{32}Br_2S_2$ ($[M]^+$): 806.0307. Found: 806.0312.

1,7-Bis[2-(10-bromo-9-anthryl)ethynyl)-3-thienyl]heptane (4b): According to the general procedure **II**: **3b** (1.56 g, 4.99 mmol), 9-bromo-10-iodoanthracene (3.82 g, 9.97 mmol), $Pd(PPh_3)_4$ (289 mg, 0.250 mmol) and CuI (47 mg, 0.25 mmol) in diisopropylamine (10 mL) and toluene (30 mL) to give **4b** (3.60 g, 4.38 mmol) in 88% as a yellow solid: Mp.: 191–192 °C; ^1H NMR (400 MHz, $CDCl_3$): δ 8.40–8.60 (m, 8H), 7.49–7.62 (m, 8H), 7.27 (d, J= 5.2 Hz, 2H), 6.89 (d, J= 5.2 Hz, 2H), 2.88 (t, J= 7.6 Hz, 4H), 1.69–1.81 (m, 4H), 1.45–1.58 (m, 6H); ^{13}C NMR (100 MHz, $CDCl_3$): δ 148.0, 132.5, 130.2, 128.5, 128.2, 127.3, 127.0, 126.72, 126.69, 123.9, 118.5, 118.2, 94.8, 92.0, 30.5, 29.9, 29.3, 29.1; HRMS (APCI, positive) calculated for $C_{47}H_{34}Br_2S_2$ ($[M]^+$): 820.0463. Found:

820.0473.

Macrocyclic dimer 1a: According to the general procedure **III**: **3a** (267 mg, 0.895 mmol), **4a** (710 mg, 0.878 mmol), $Pd(PPh_3)_4$ (50.8 mg, 0.0440 mmol) and CuI (8.3 mg, 0.044 mmol,) in diisopropylamine (30 mL) and THF (100 mL). Purification by flash column chromatography (CH_2Cl_2/hexane = 1:2, R_f = 0.40) gave **1a** (124 mg, 0.131 mmol) in 15% as an orange solid: Mp. > 280 °C; ^{1}H NMR (400 MHz, $CDCl_3$): δ 8.30–8.38 (m, 8H), 7.27–7.39 (m, 12H), 6.99 (d, J= 5.2 Hz, 4H), 2.96 (t, J= 7.6 Hz, 8H), 1.80–1.92 (m, 8H), 1.59–1.68 (m, 8H); ^{13}C NMR (100 MHz, $CDCl_3$): δ 147.7, 131.4, 128.5, 126.8, 126.7, 126.6, 118.9, 118.0, 95.1, 92.8, 30.5, 30.1, 29.2; HRMS (APCI, positive) calculated for $C_{64}H_{49}S_4$ ([M+H]$^+$): 945.2712. Found: 945.2717.

Macrocyclic dimer 1b: According to the general procedure **III**: **3a** (152 mg, 0.509 mmol), **4b** (410 mg, 0.498 mmol), $Pd(PPh_3)_4$ (29.0 mg, 0.0251 mmol) and CuI (4.7 mg, 0.025 mmol) in diisopropylamine (10 mL) and THF (90 mL). Purification by flash column chromatography (CH_2Cl_2/hexane = 1:2, R_f = 0.40) gave **1b** (86 mg, 0.090 mmol) in 18% as a deep orange solid: Mp. > 280 °C; ^{1}H NMR (400 MHz, $CDCl_3$): δ 8.30–8.39 (m, 8H), 7.34–7.42 (m, 8H), 7.28–7.34 (m, 4H), 6.97–7.01 (m, 4H), 2.90–3.01 (m, 8H), 1.78–1.93 (m, 8H), 1.62–1.68 (m, 4H), 1.52–1.62 (m, 4H); ^{13}C NMR (100 MHz, $CDCl_3$): δ 147.8, 147.7, 131.34, 131.30, 128.54, 128.51, 126.85, 126.83, 126.71, 126.65, 126.59, 118.9, 118.0, 117.9, 95.2, 95.1, 92.9, 92.8, 30.7, 30.6, 30.1, 30.0, 29.5, 29.3, 29.1; HRMS (APCI, positive) calculated for $C_{65}H_{51}S_4$ ([M+H]$^+$): 959.2868. Found: 959.2872.

Macrocyclic dimer 1c: According to the general procedure **III**: **3b** (95.0 mg, 0.304 mmol), **4b** (246 mg, 0.299 mmol), $Pd(PPh_3)_4$ (17.3 mg, 0.0150 mmol,) and CuI (2.8 mg, 0.015 mmol,) in diisopropylamine (5 mL) and THF (60 mL). Purification by flash column chromatography (CH_2Cl_2/hexane = 1:2, R_f = 0.40) gave **1c** (60 mg, 0.616 mmol) in 21% as a deep orange solid: Mp. > 280 °C; ^{1}H NMR (400 MHz, $CDCl_3$): δ 8.26–8.32 (m, 8H), 7.36–7.43 (m, 8H), 7.33 (d, J= 5.2 Hz,

4H), 7.00 (d, J = 5.2 Hz, 4H), 2.93 (t, J = 7.6 Hz, 8H), 1.77–1.94 (m, 8H), 1.54–1.68 (m, 8H); ^{13}C NMR (100 MHz, $CDCl_3$): δ 147.7, 131.1, 128.5, 126.8, 126.7, 126.5, 119.0, 117.8, 95.1, 93.0, 30.8, 30.0, 29.5, 29.2; HRMS (APCI, positive) calculated for $C_{66}H_{52}S_4$ ($[M]^+$): 972.2946. Found: 972.2956.

II. Crystallographic data, mechanical bending behavior, SEM images, powder X-ray diffraction patterns, and DSC profiles

The structure was solved by direct methods (SHELXS-97 or SHELXS-2013)[3,4] and refined by least-squares calculations on F^2 for all independent reflections (SHELXL-97 or SHELXL-2013). All non-hydrogen atoms were refined with anisotropic displacement parameters, and hydrogen atoms were placed in idealized positions and refined by applying riding models with the relative isotropic displacement parameters. Crystal morphology calculation of **1a** and **1b** based on their crystal structures using the Bravais-Friedel-Donnay-Harker (BFDH) method was conducted by the Morphology module in the Materials Studio 7.0 simulation package distributed by Accelrys Inc., San Diego CA (2014).

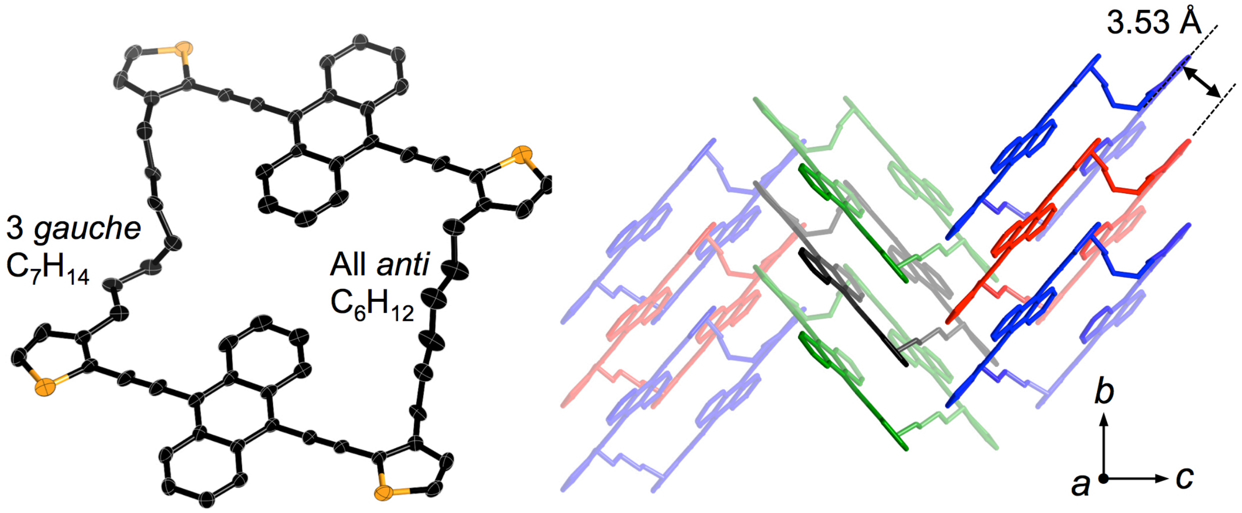

Fig. S1. X-ray crystal structure of **1b**. Molecular structure (left) and packing structure (right). Thermal ellipsoids are drawn at the 50% probability level.

[3] G. M. Sheldrick, *Acta Cryst.,* 2008, **A64**, 112.

[4] T. Gruene, H. W. Hahn, A. V. Luebben, F. Meilleurb and G. M. Sheldrick, *J. Appl. Cryst.,* 2014, **47**, 462.

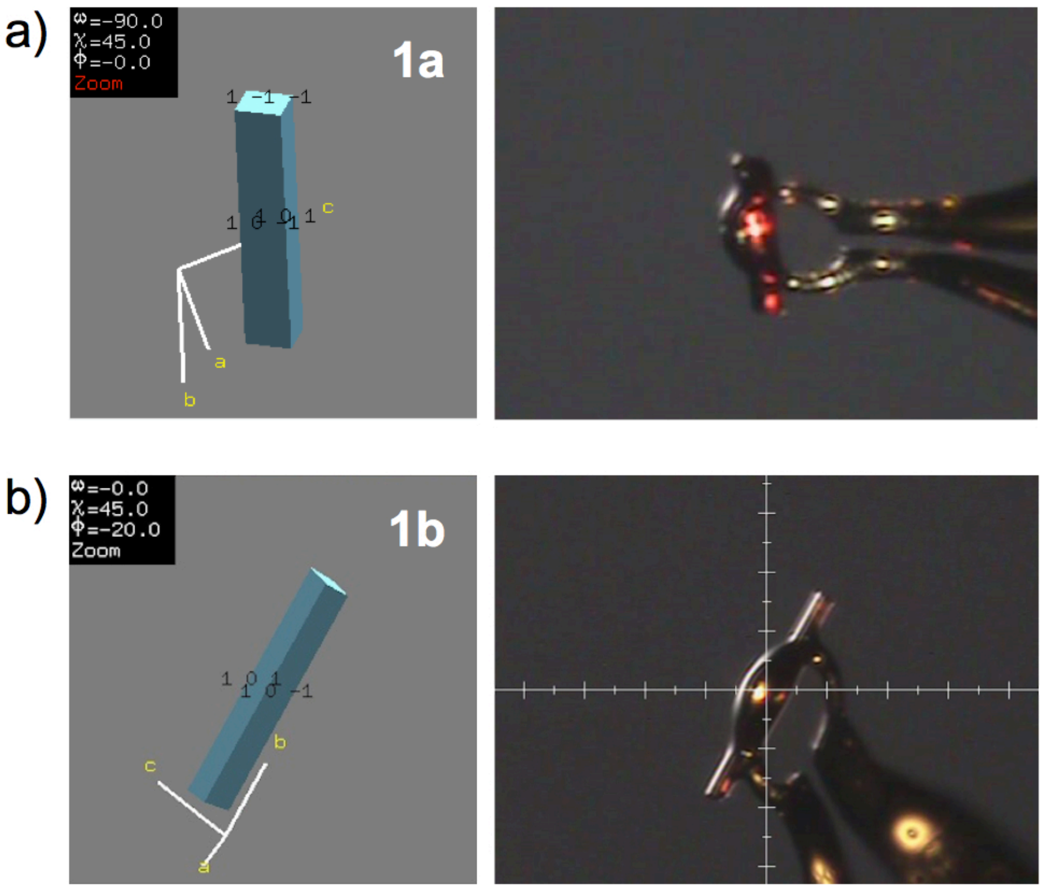

Fig. S2. Face index analysis of **1a** and **1b** based on the X-ray crystallography.

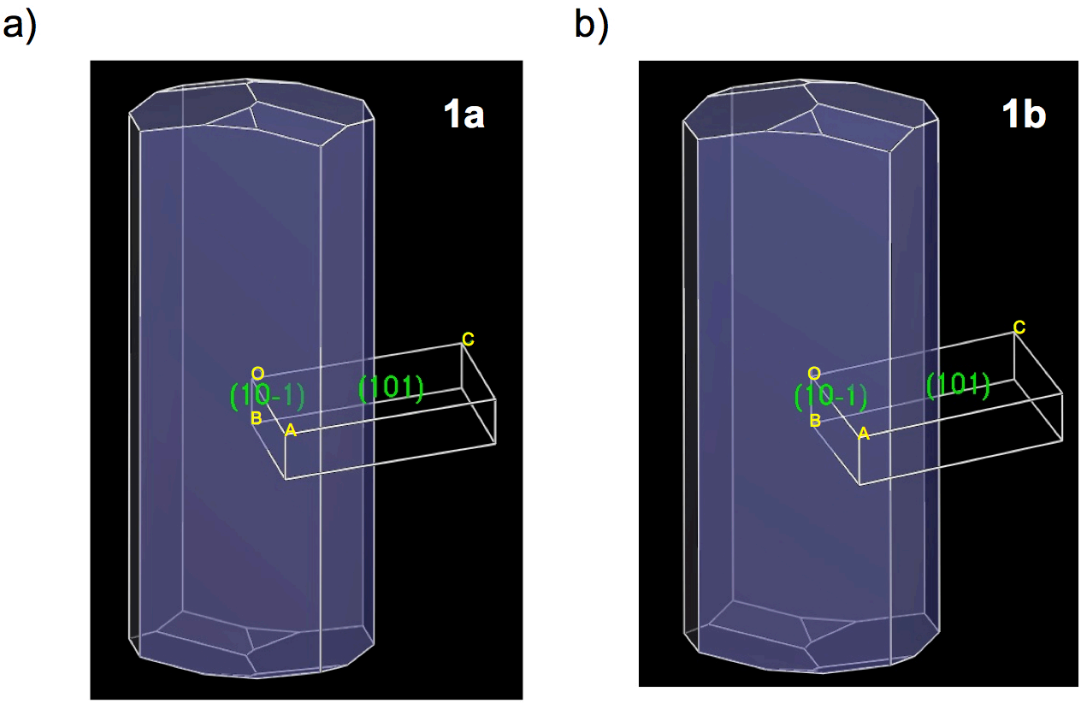

Fig. S3. Crystal morphology calculation of **1a** and **1b** using the Bravais-Friedel-Donnay-Harker (BFDH) method.

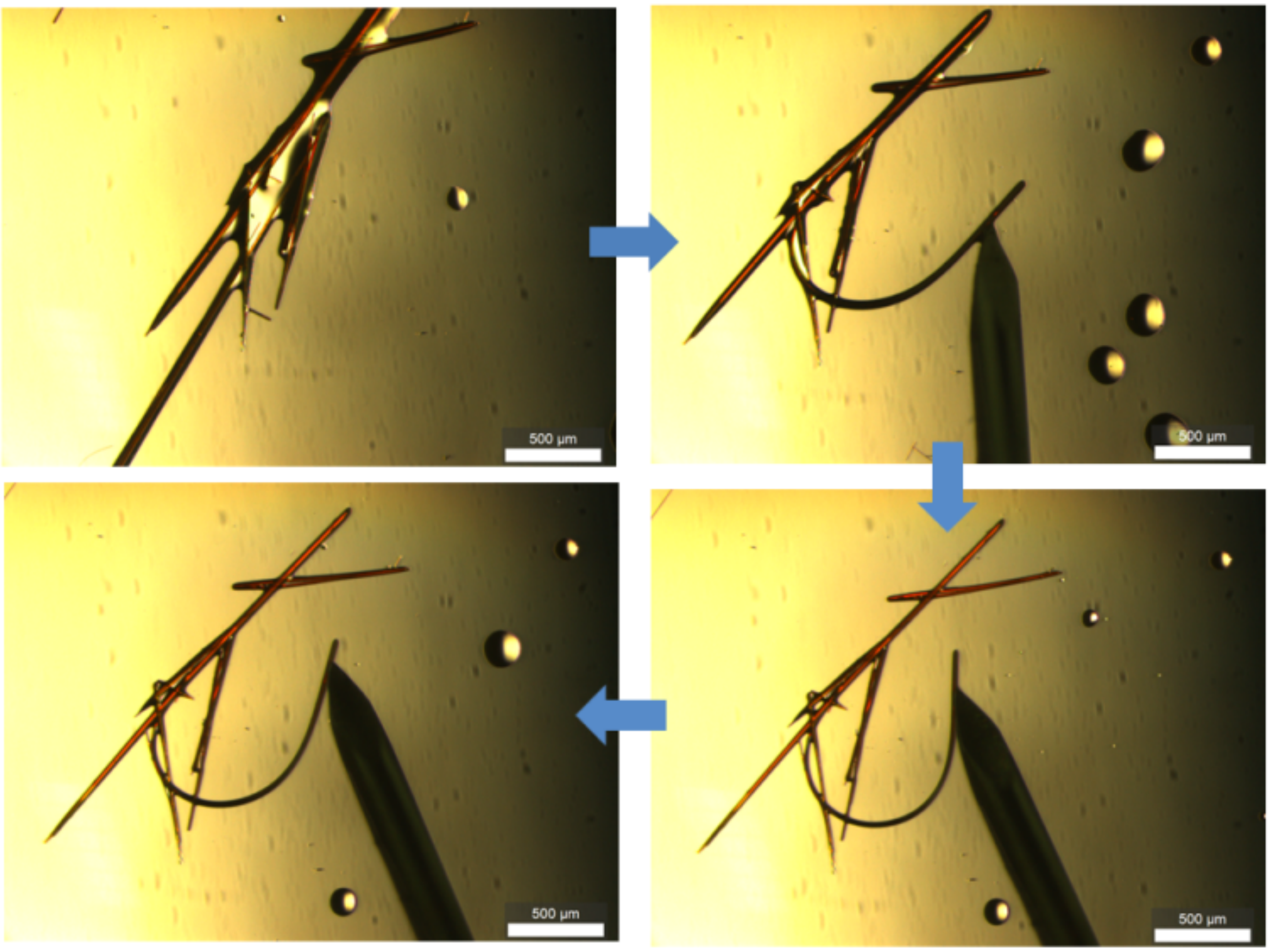

Fig. S4. Mechanical bending of macrocyclic dimer **1b** by a metallic pin at ambient temperature. The scale bar represents 500 μm.

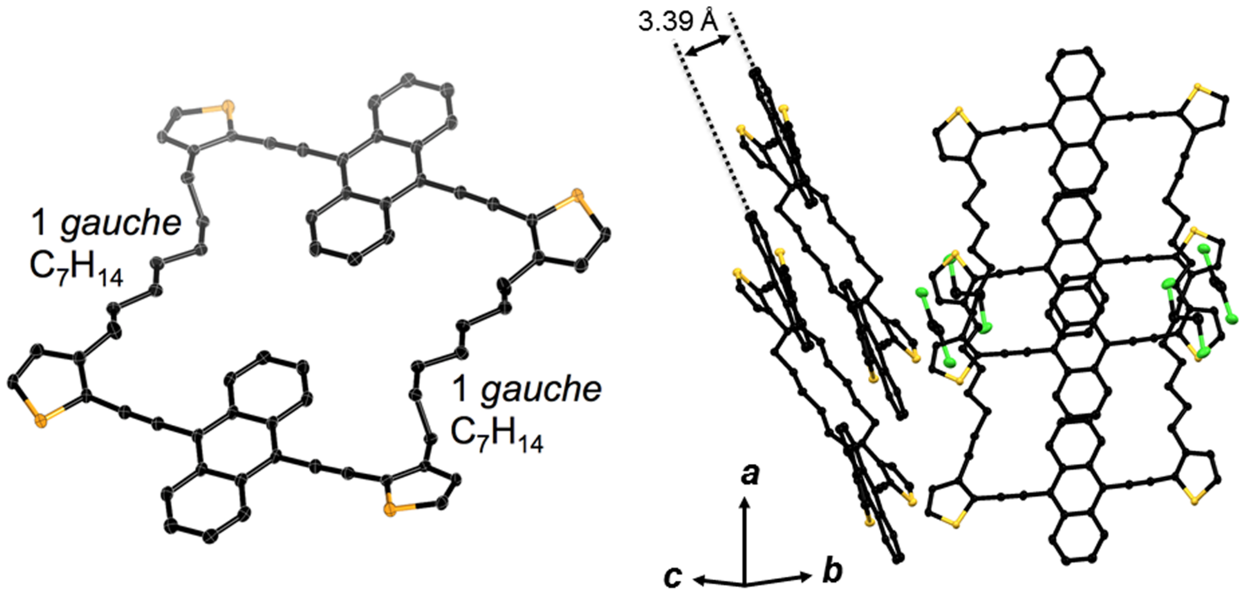

Figure S5. X-ray crystal structure of **1c·prism**. Molecular structure (left) and crystal packing (right). Thermal ellipsoids are drawn at the 50% probability level.

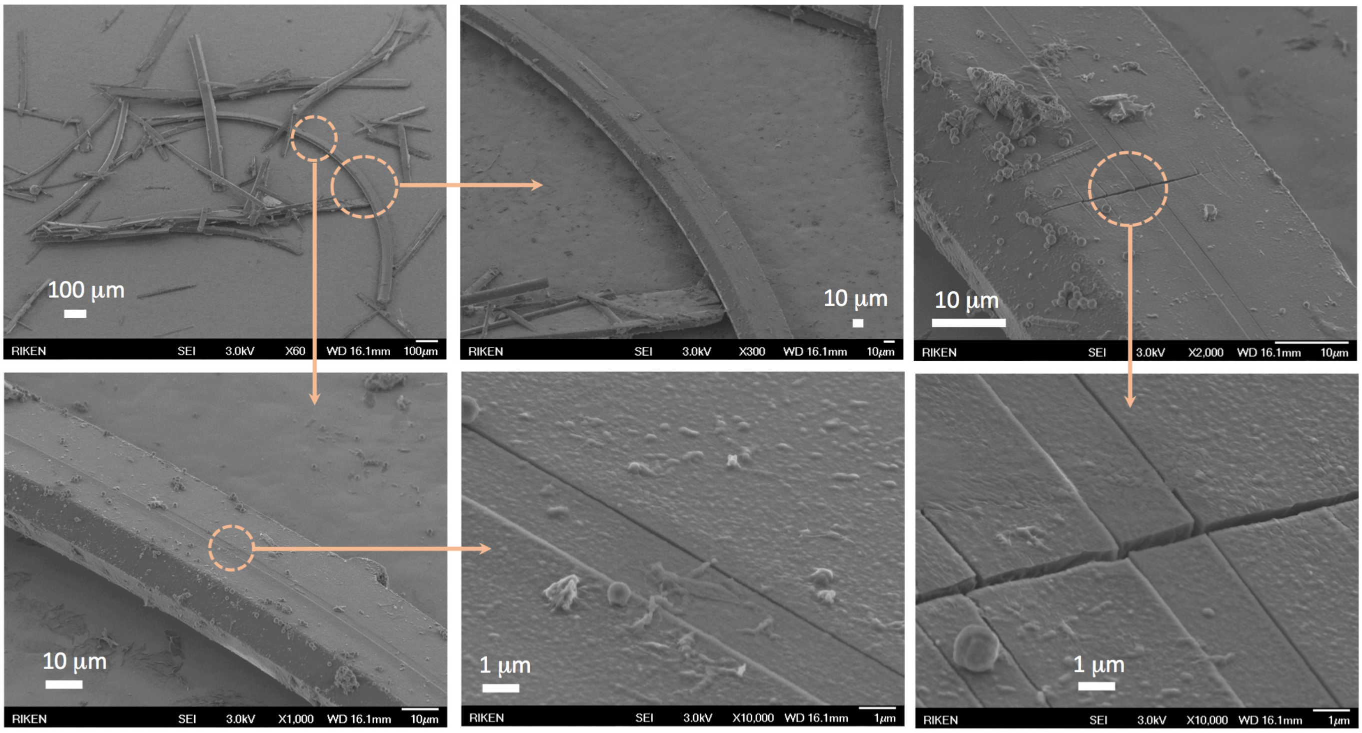

Fig. S6. Scanning electron micrograph of **1c·bent**.

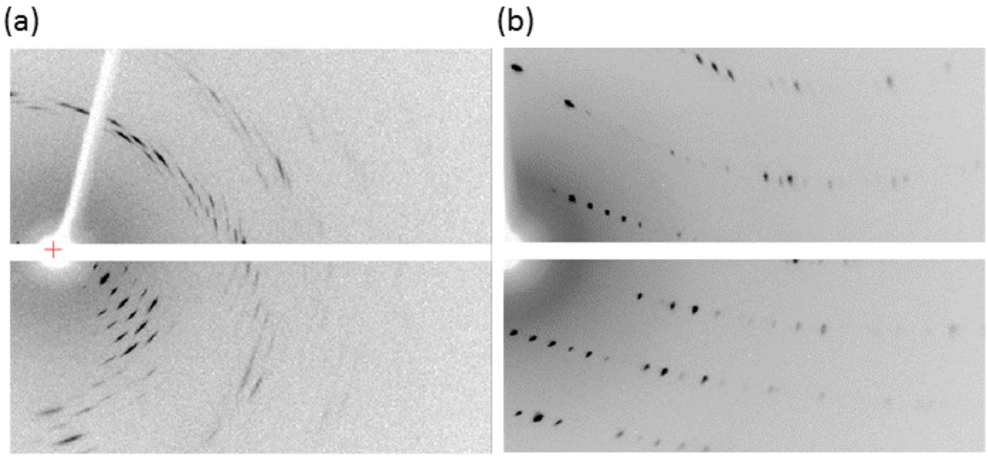

Fig. S7. Observed diffraction spots of **1c·bent** a) before and b) after the annealing process. The straight part of the crystal, which was cut into small pieces, was used for the X-ray diffraction experiment.

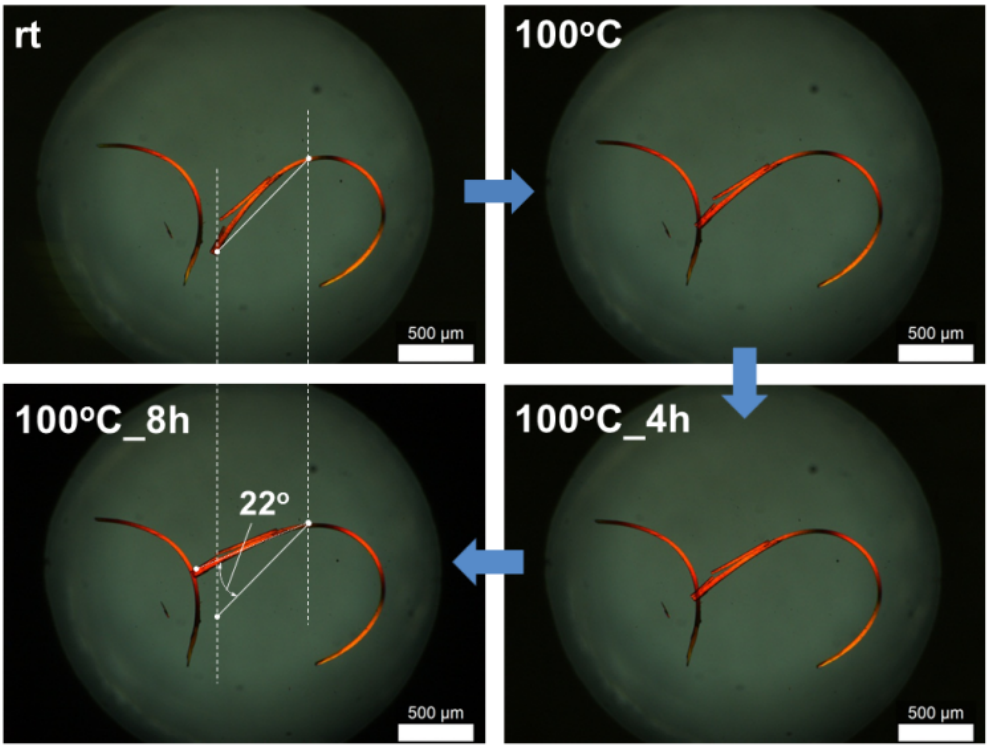

Fig. S8. Macroscopic motion during the annealing process of **1c·bent** at 100 °C.

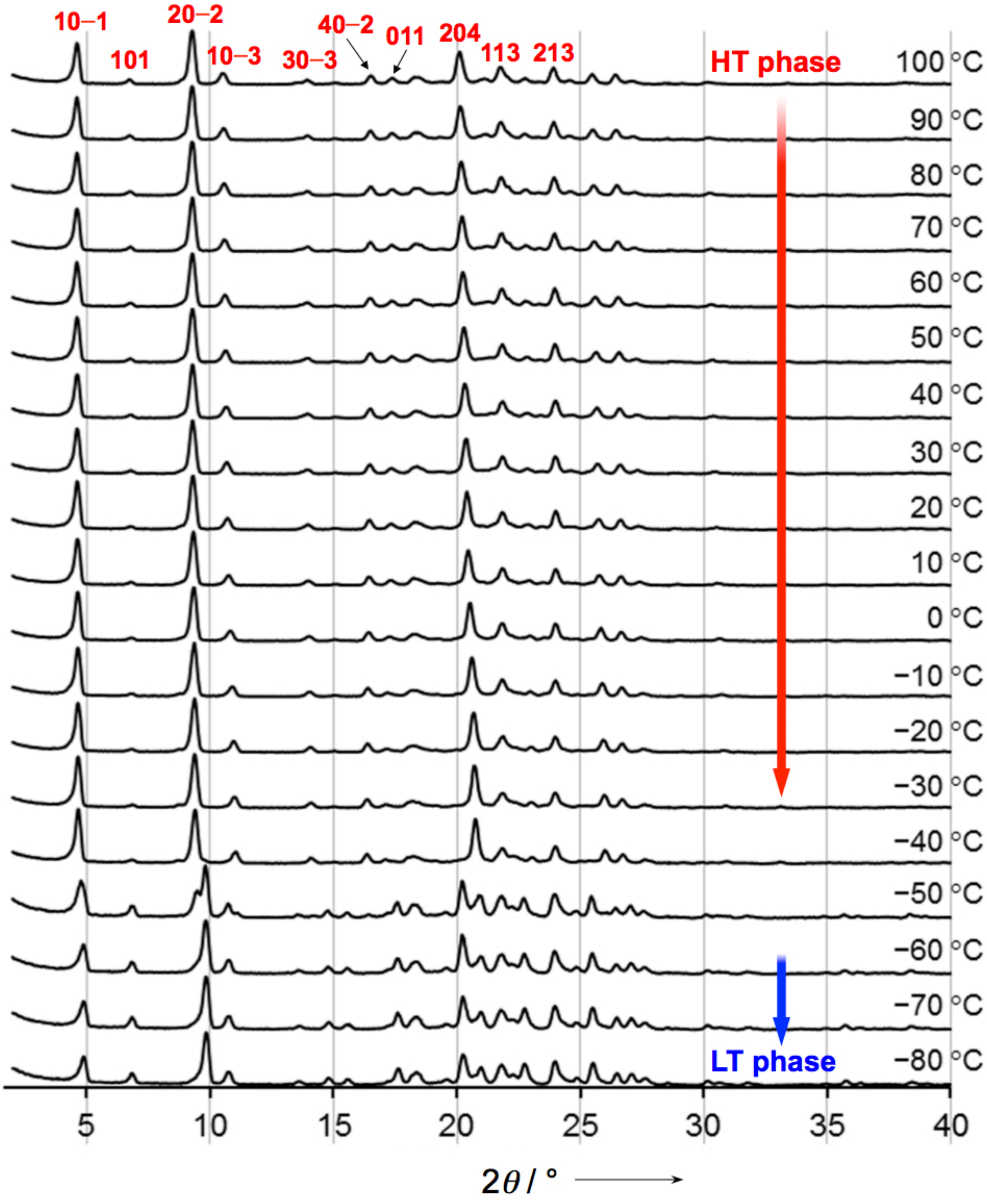

Fig. S9. Powder X-ray diffraction patterns of **1c·bent** in the first cooling process from 100 °C to −80 °C, in which Cu $K\alpha$ radiation was used.

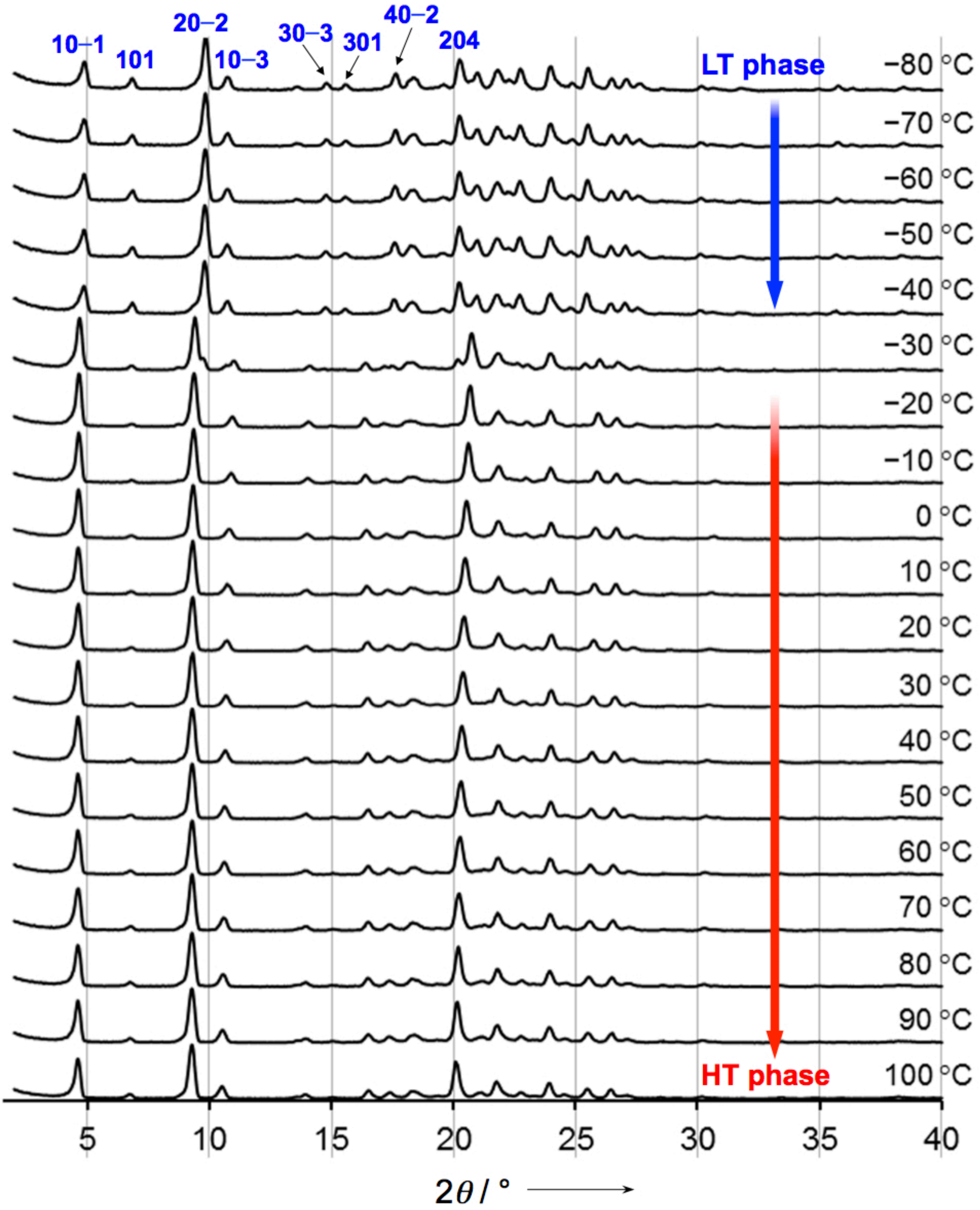

Fig. S10. Powder X-ray diffraction patterns of **1c·bent** in the second heating process from −80 °C to 100 °C, in which Cu *K*α radiation was used.

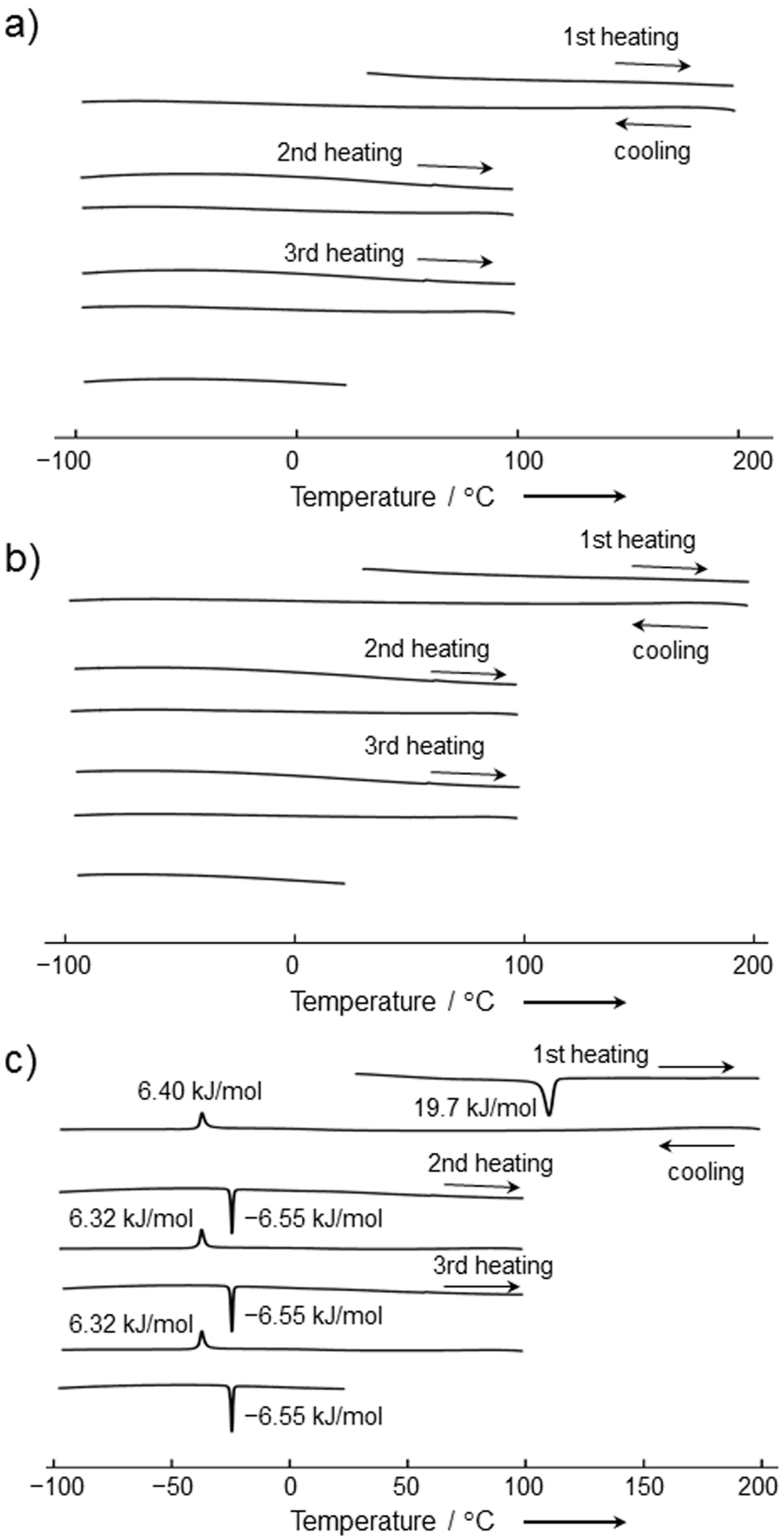

Fig. S11. DSC profiles of **1a**, **1b**, and **1c·bent**.

Table S1. Crystallographic Data of **1c·prism** and **1c·bent** Before the Annealing Process

	**1c·prism**	**1c·bent** (before annealing)
**Formula**	$C_{66}H_{52}S_4{\cdot}C_2H_4Cl_2$	$C_{66}H_{52}S_4$
T **(°C)**	−170	20
**Crystal System**	monoclinic	monoclinic
**Space Group**	$P2_1/c$	$P2_1/n$
a **(Å)**	7.6706(11)	21.62(8)
b **(Å)**	17.4871(15)	5.283(16)
c **(Å)**	20.506(3)	24.82(8)
β **(°)**	100.272(5)	111.57(7)
V **(Å³)**	2706.5(6)	2637(15)
Z	2	2
**GOF**	1.116	1.356
R_{int}	0.0220	0.0497
R(F)	0.0376	0.1070
$wR(F^2)$	0.1202	0.3841
CCDC	1013994	–

Table S2. Crystal Lattices Determined by X-ray Crystallographic Analysis at Various Temperatures[a]

Temp / °C	*a* / Å	*b* / Å	*c* / Å	*β* / °	Cell volume / $Å^3$
20	21.52	5.25	24.81	111.8	2599
0	21.54	5.29	24.60	111.8	2598
−20	21.60	5.31	24.31	111.9	2587
−40[b]	–	–	–	–	–
−60	20.21	5.27	24.70	109.1	2485
−80	20.19	5.26	24.66	109.2	2475

[a] The crystal was annealed at 100 °C for 30 min and then slowly cooled down to corresponding temperatures ($\Delta T/\Delta t$ = 2 °C/min). The temperature was kept for 5 min before the measurements. [b] The lattice parameter at −40 °C was not able to be determined probably due to the serious conformational heterogeneity in the transition period.

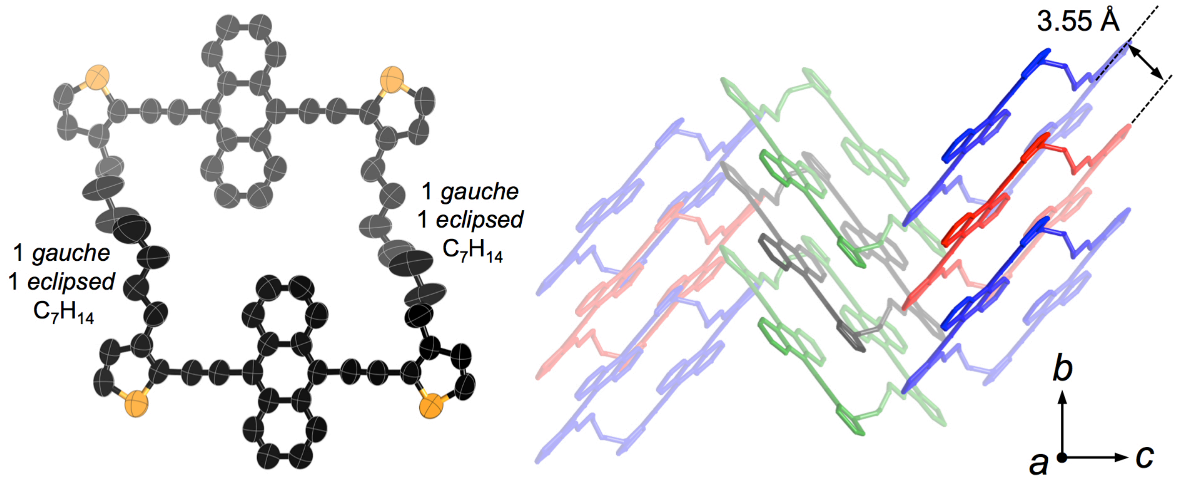

Fig. S12. X-ray crystal structure of **1c·bent** at 20 °C. Molecular structure (left) and crystal packing (right). Thermal ellipsoids are drawn at the 50% probability level.

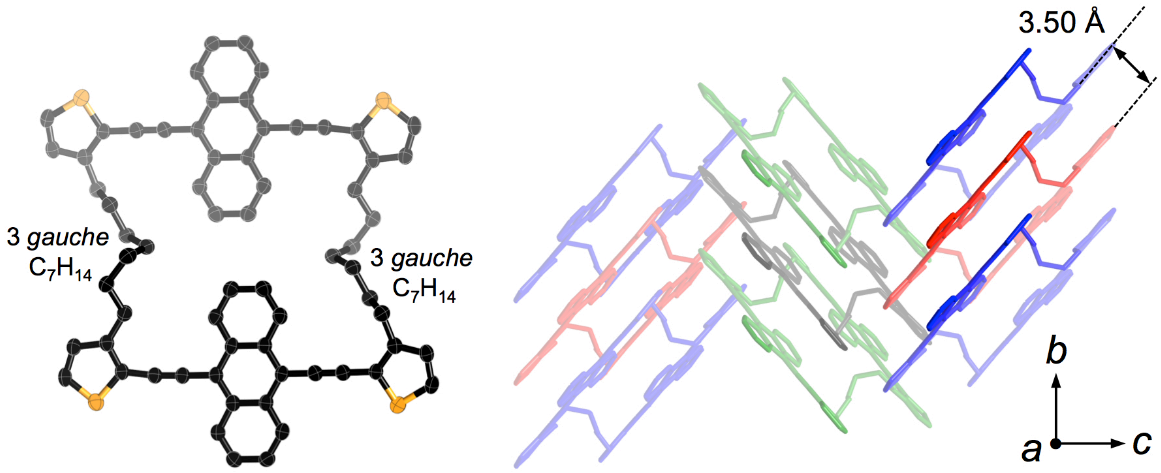

Fig. S13. X-ray crystal structure of **1c·bent** at −150 °C. Molecular structure (left) and crystal packing (right). Thermal ellipsoids are drawn at the 50% probability level.

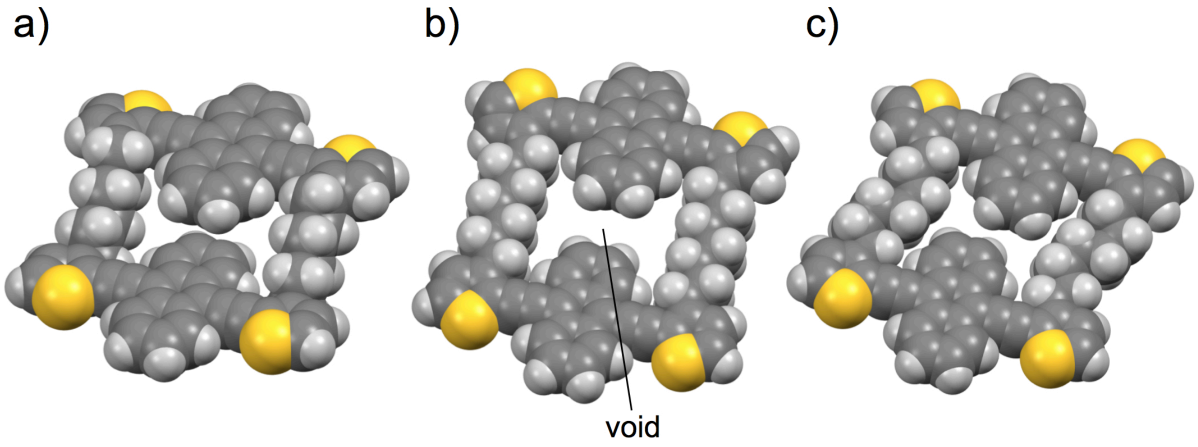

Fig. S14. Space filling representation for a) the crystal structure of **1a**, b) a modeled structure **1c** with heptylene linkers taking all-*anti* conformation, and c) the crystal structure of **1c·bent** at 20 °C.

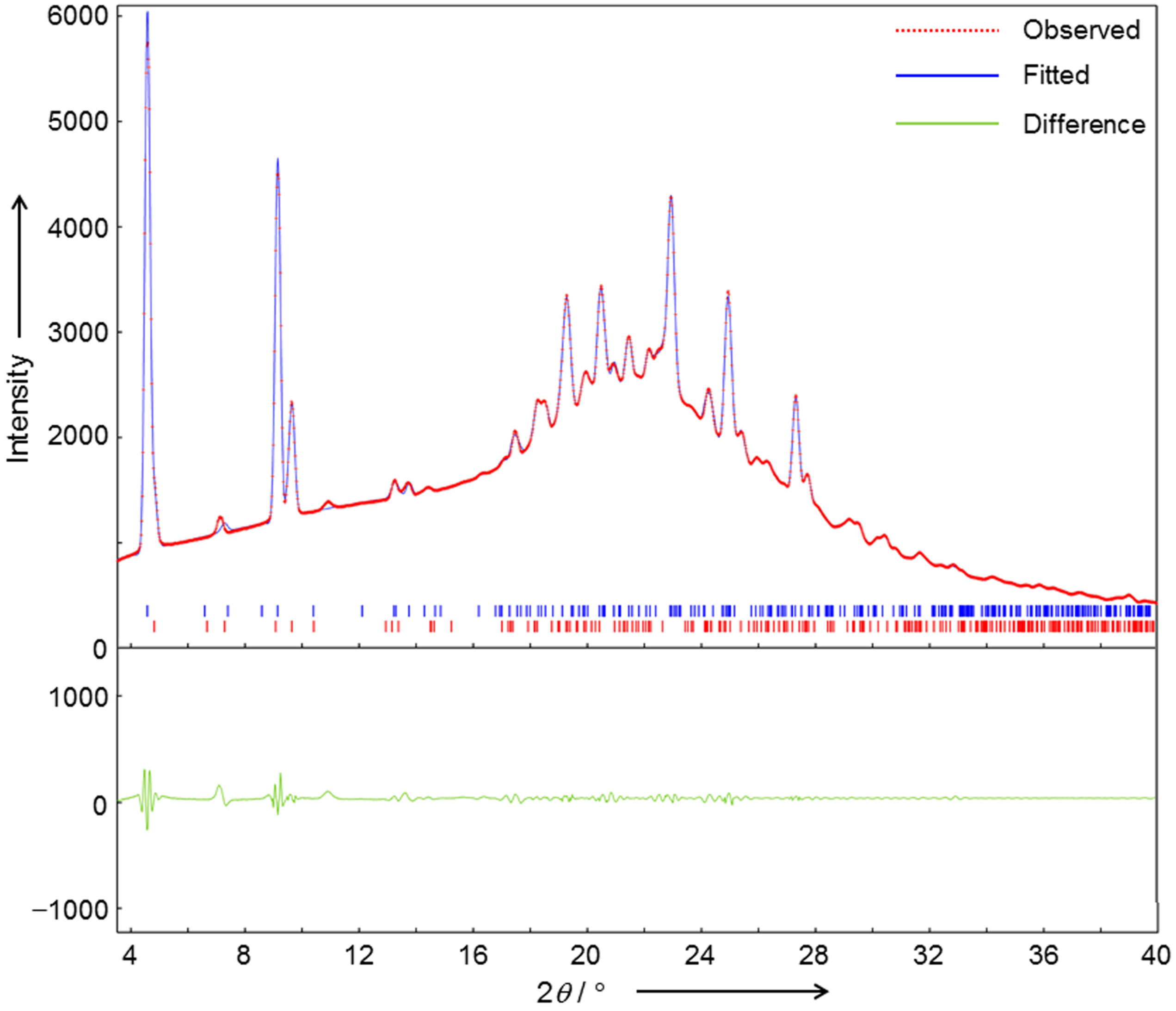

Fig. S15. Observed powder X-ray diffraction pattern (red-dotted line) of freshly prepared **1c·bent** crystal before annealing and its fitted pattern (blue line) as a mixture of the HT and LT phases (R_{wp} = 0.0142, R_p = 0.0080, R_e = 0.0235, S = 0.6027). The refined cell parameters were shown in Table S3. Green line demonstrates the difference between the observed and fitted patterns. The X-ray diffraction measurement was performed at room temperature (*ca.* 20 °C), in which Cu $K\alpha$ radiation was used.

Table S3. Refined crystal lattice parameters of freshly prepared **1c·bent** crystal fitted by Pawley method.

Crystal phase	a / Å	b / Å	c / Å	β / °	V / Å^3
HT	21.821(11)	5.381(2)	25.329(13)	110.77(3)	2781(2)
LT	20.414(13)	5.303(3)	25.408(16)	108.78(3)	2604(3)

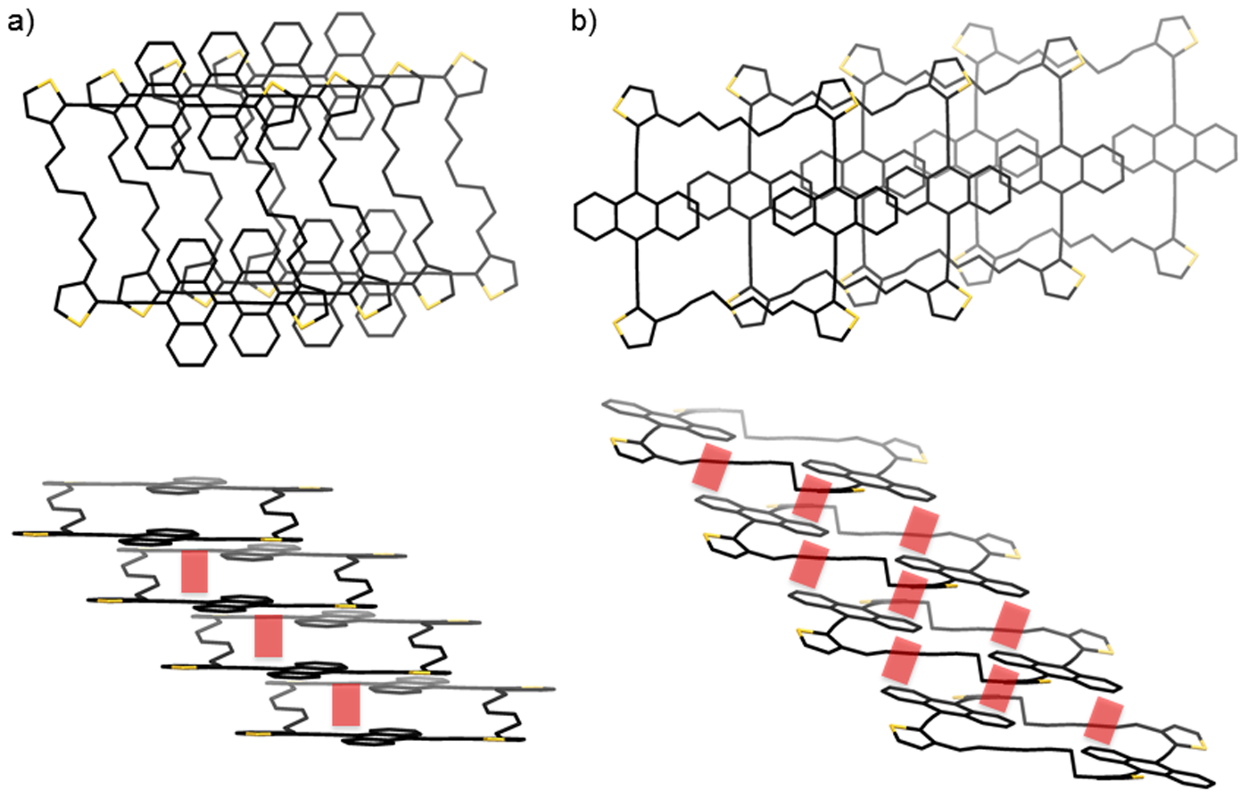

Fig. S16. Top and side views of the crystal packing structures in a) **1c·bent HT** and b) **1c·prism**. Hydrogen atoms are omitted for clarity. Red squares demonstrate the interactions between the π stacking moieties.

The orientation of the π stacking moieties in **1c·prism** is different from those in **1a**, **1b**, and **1c·bent**. In the crystal packing of **1c·prism**, the macrocycles are slipped in the lateral direction of the bis(thienylethynyl)anthracene moieties, while those are slipped in the longitudinal direction in the other crystals (Fig. S16). As a result, an anthracene moiety in **1c·prism** is stacked with three adjacent anthracene moieties, though its overlap is small. The doubly layered anthracene arrays are formed, in which the dichloroethane molecules are accommodated between them. On the other hand in **1c·bent** (either in HT or LT), the single π-stacked array of the bis(thienylethynyl)anthracene moieties is formed separately. Based on these structural analyses, we propose the thermodynamic formation of **1c·prism** crystal in the slower recrystallization protocol compared to that for **1c·bent**. When the concentration of **1c** is low and thus the recrystallization process takes a long time, the packing structure of **1c** is converged to the thermodynamically more stable **1c·prism** consisting of the double π-stacked arrays, while the high concentration leads to the kinetic formation of the separated but largely overlapped single π-stacked arrays.

III. Fluorescence spectra of the crystals

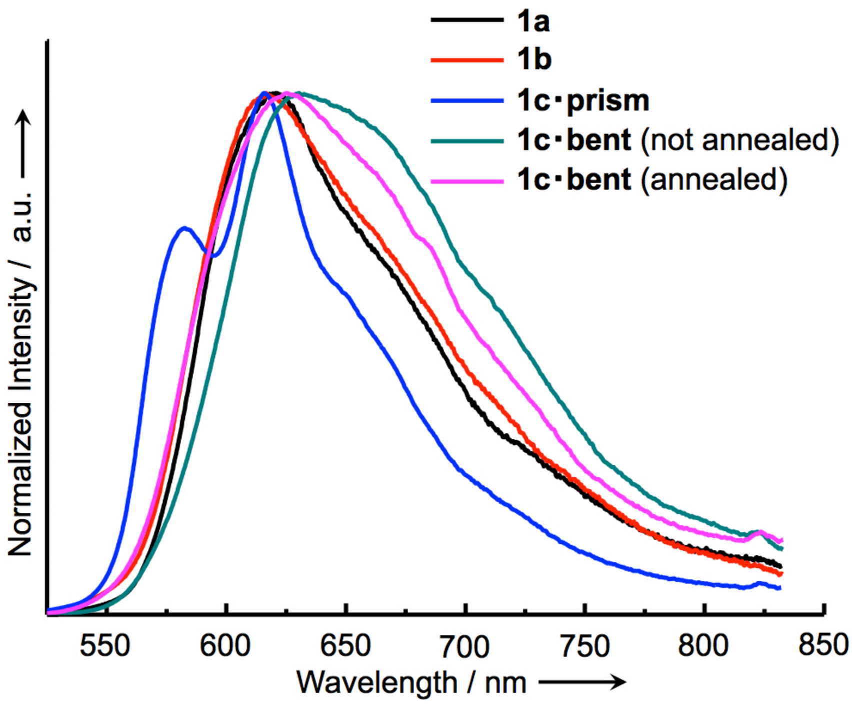

Fig. S17. Fluorescence spectra of the crystals **1a**, **1b**, **1c·prism**, and **1c·bent**.

IV. ^{1}H and ^{13}C NMR spectra

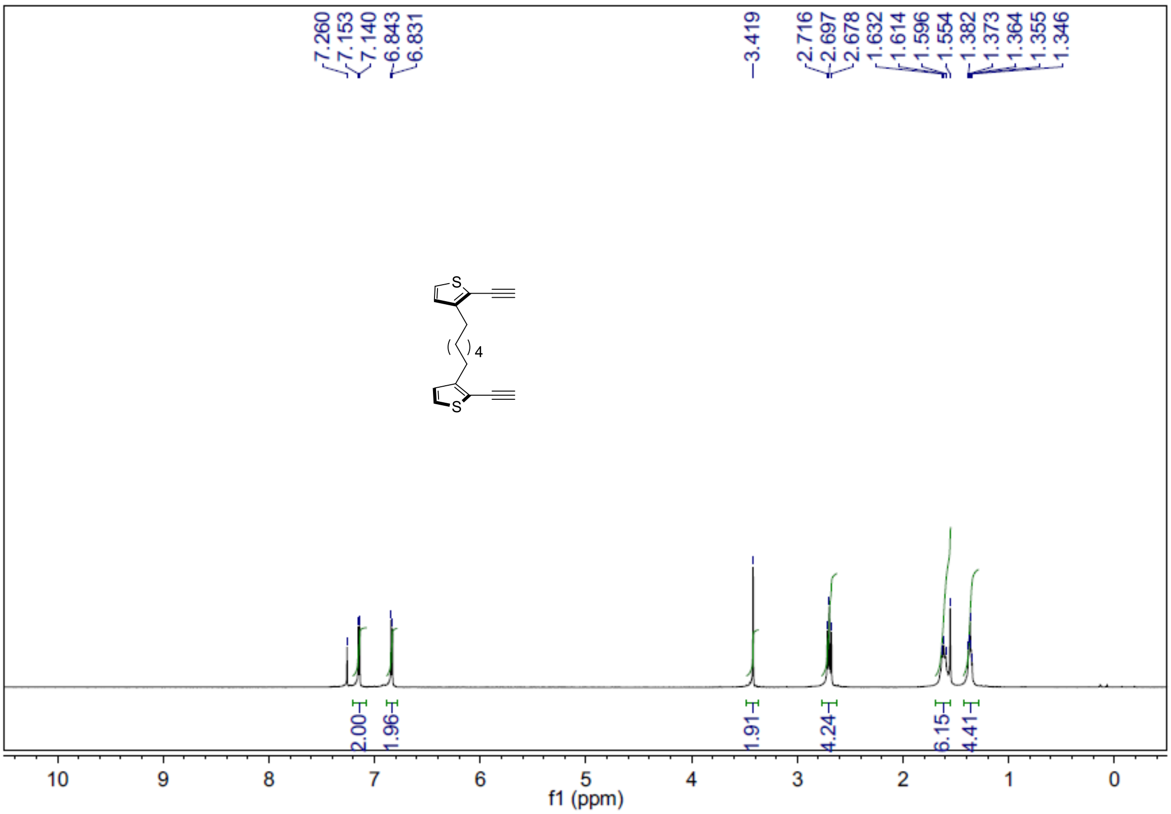

Fig. S18. ^{1}H NMR spectrum of **3a** in $CDCl_3$.

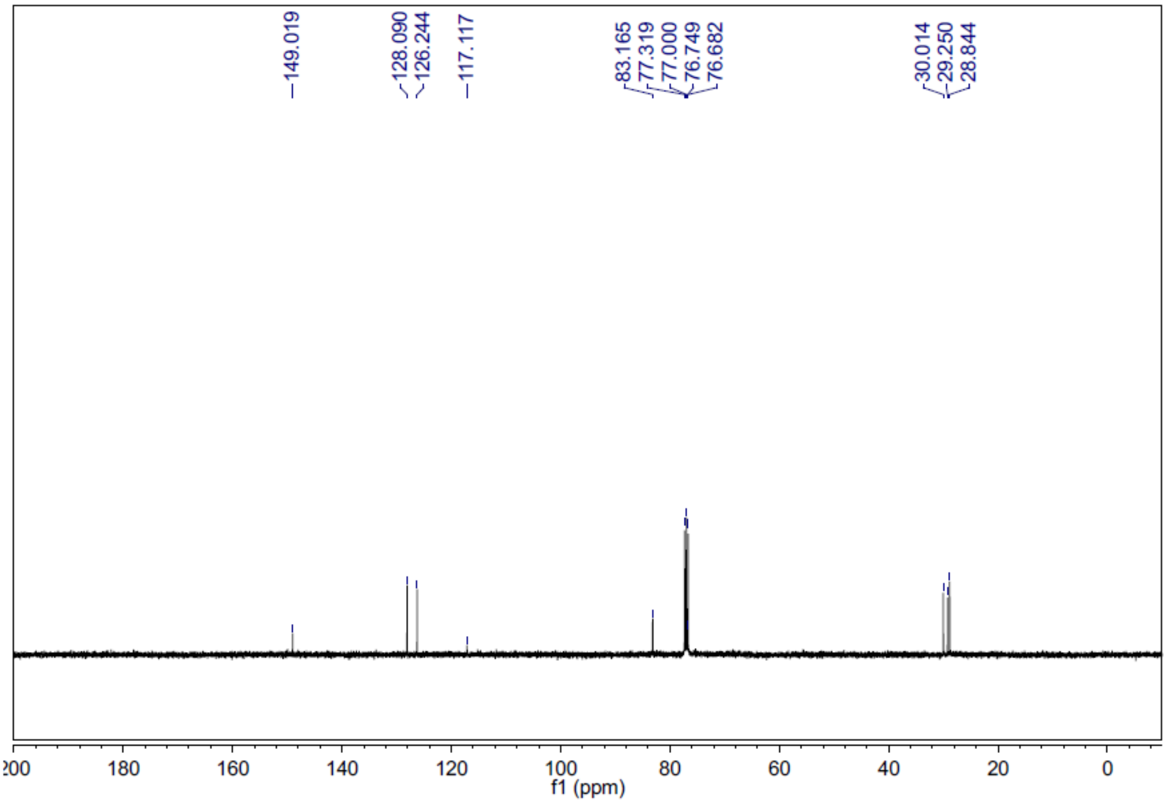

Fig. S19. ^{13}C NMR spectrum of **3a** in $CDCl_3$.

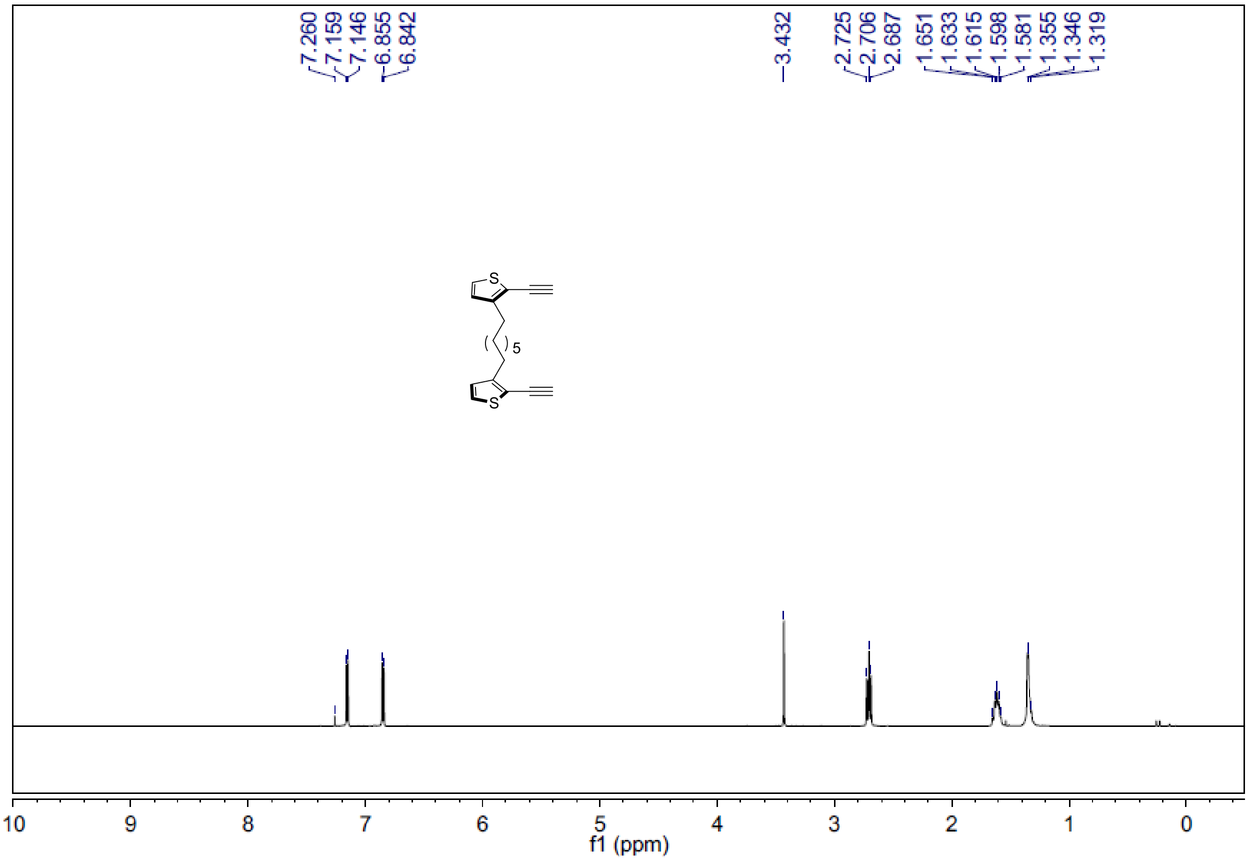

Fig. S20. ^{1}H NMR spectrum of **3b** in $CDCl_3$.

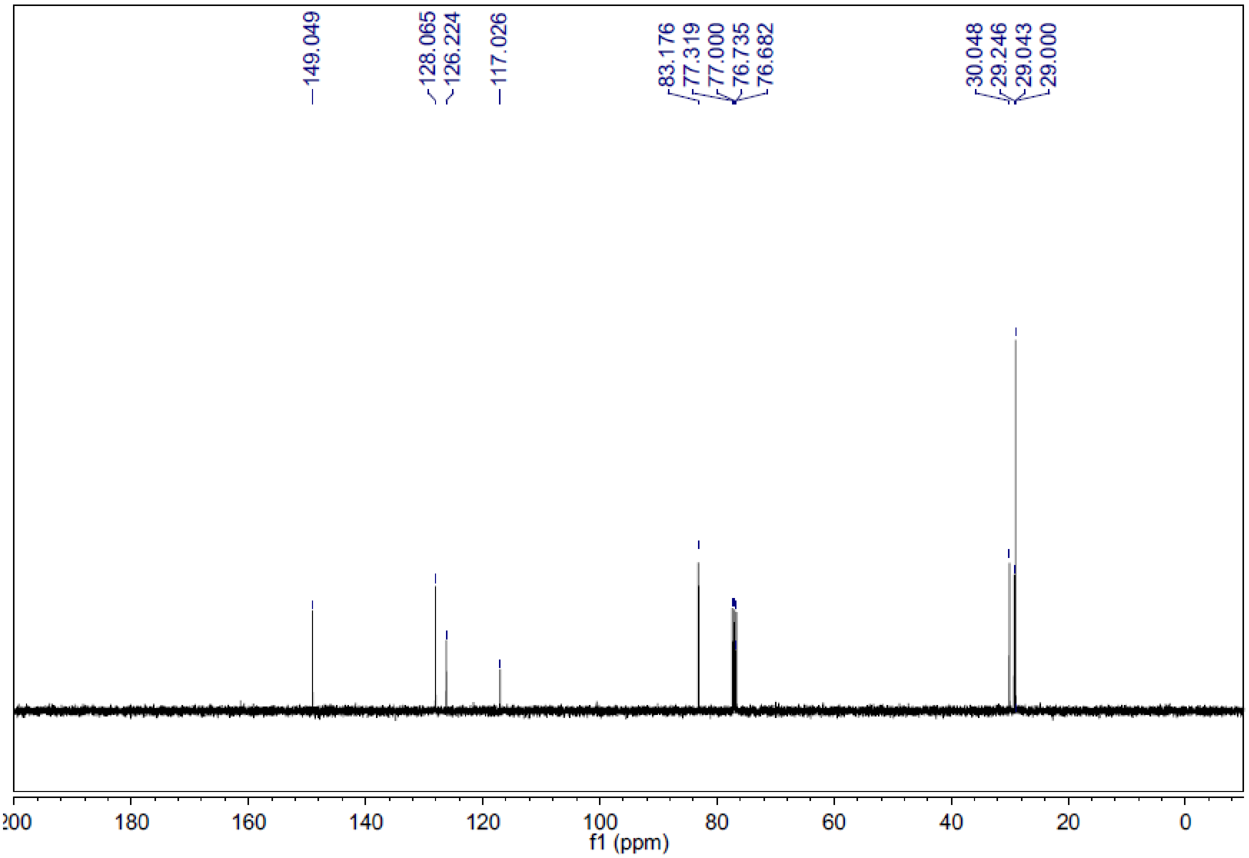

Fig. S21. ^{13}C NMR spectrum of **3b** in $CDCl_3$.

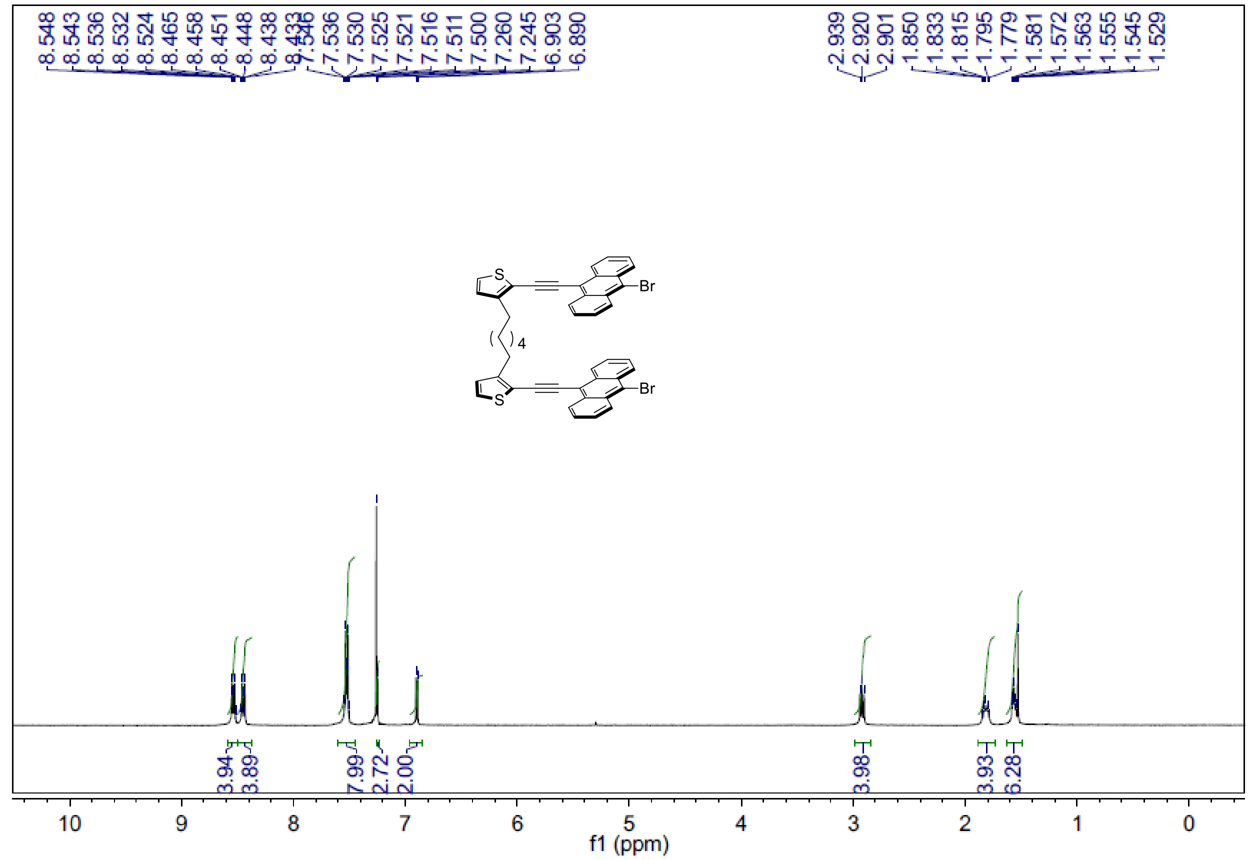

Fig. S22. ^{1}H NMR spectrum of **4a** in $CDCl_3$.

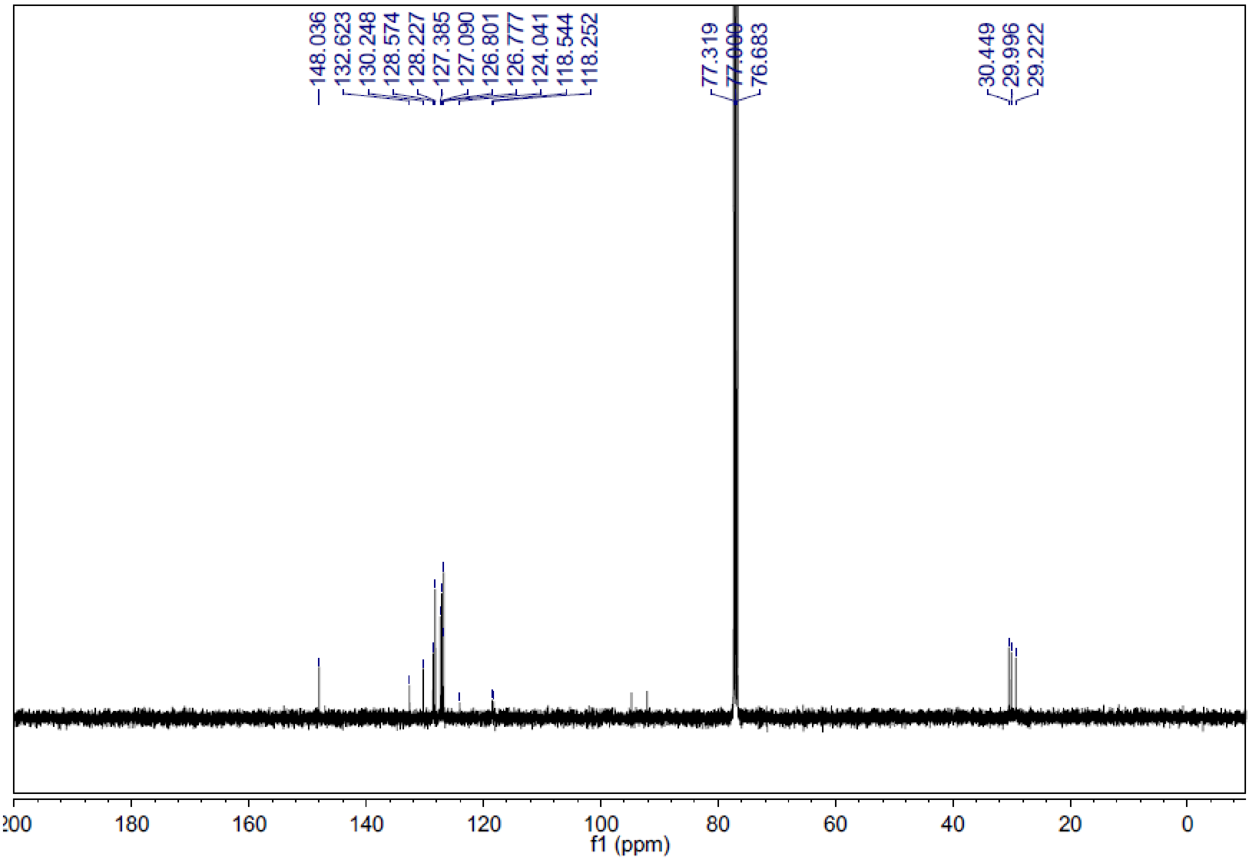

Fig. S23. ^{13}C NMR spectrum of **4a** in $CDCl_3$.

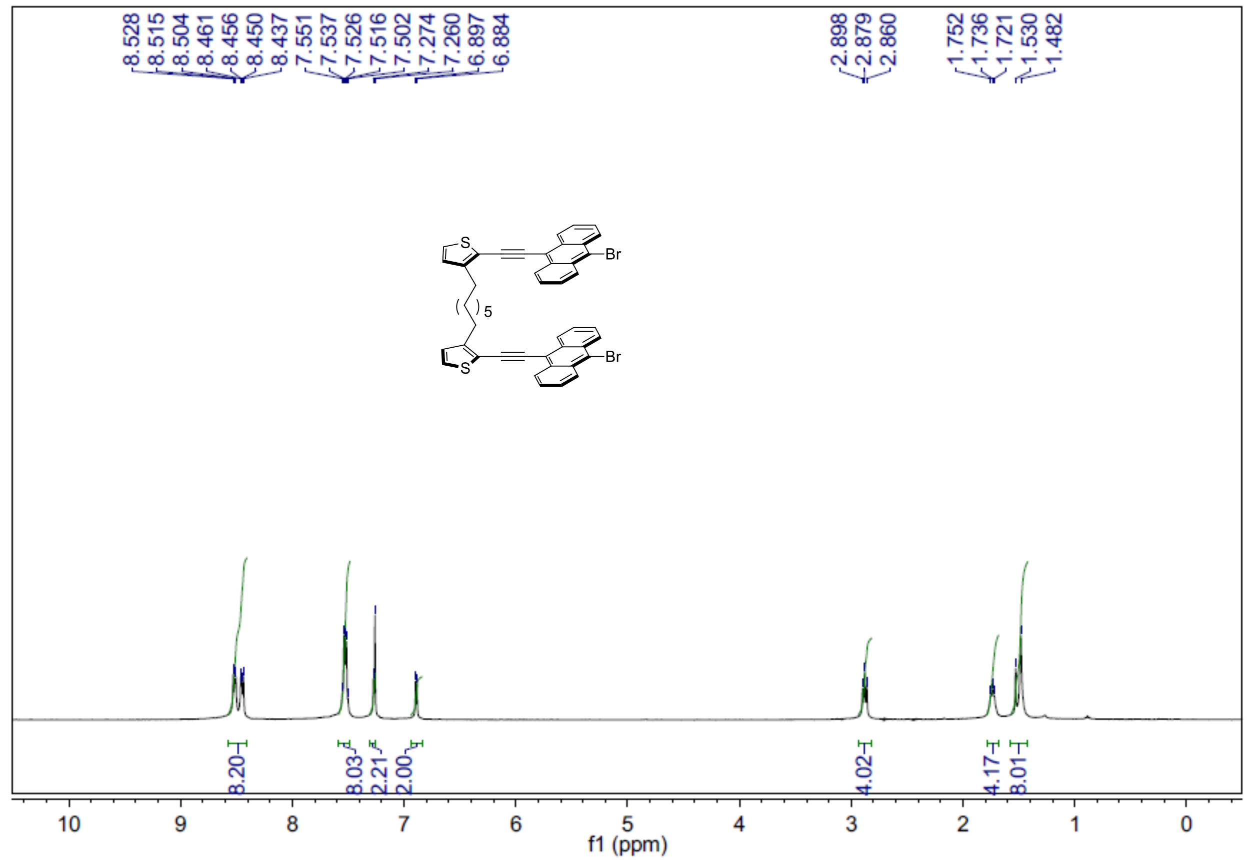

Fig. S24. ^{1}H NMR spectrum of **4b** in $CDCl_3$.

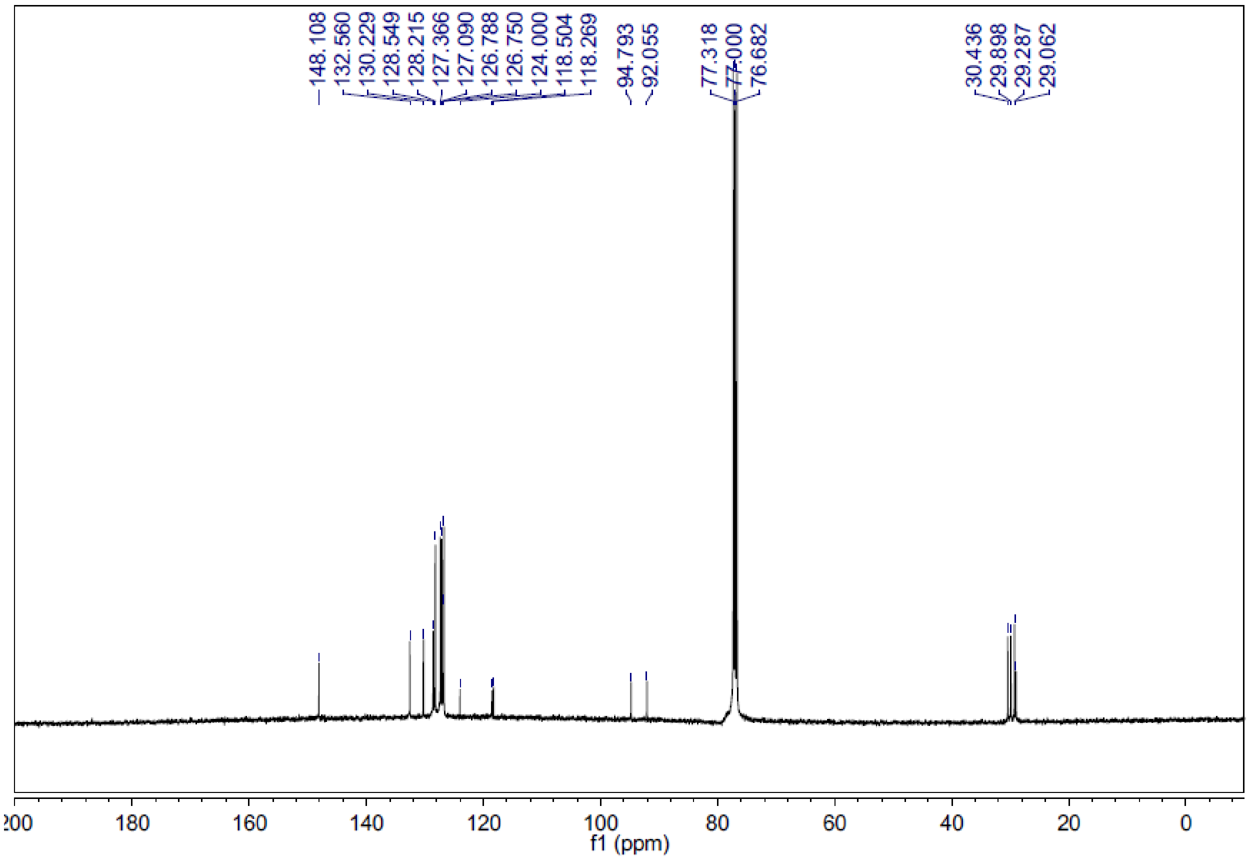

Fig. S25. ^{13}C NMR spectrum of **4b** in $CDCl_3$.

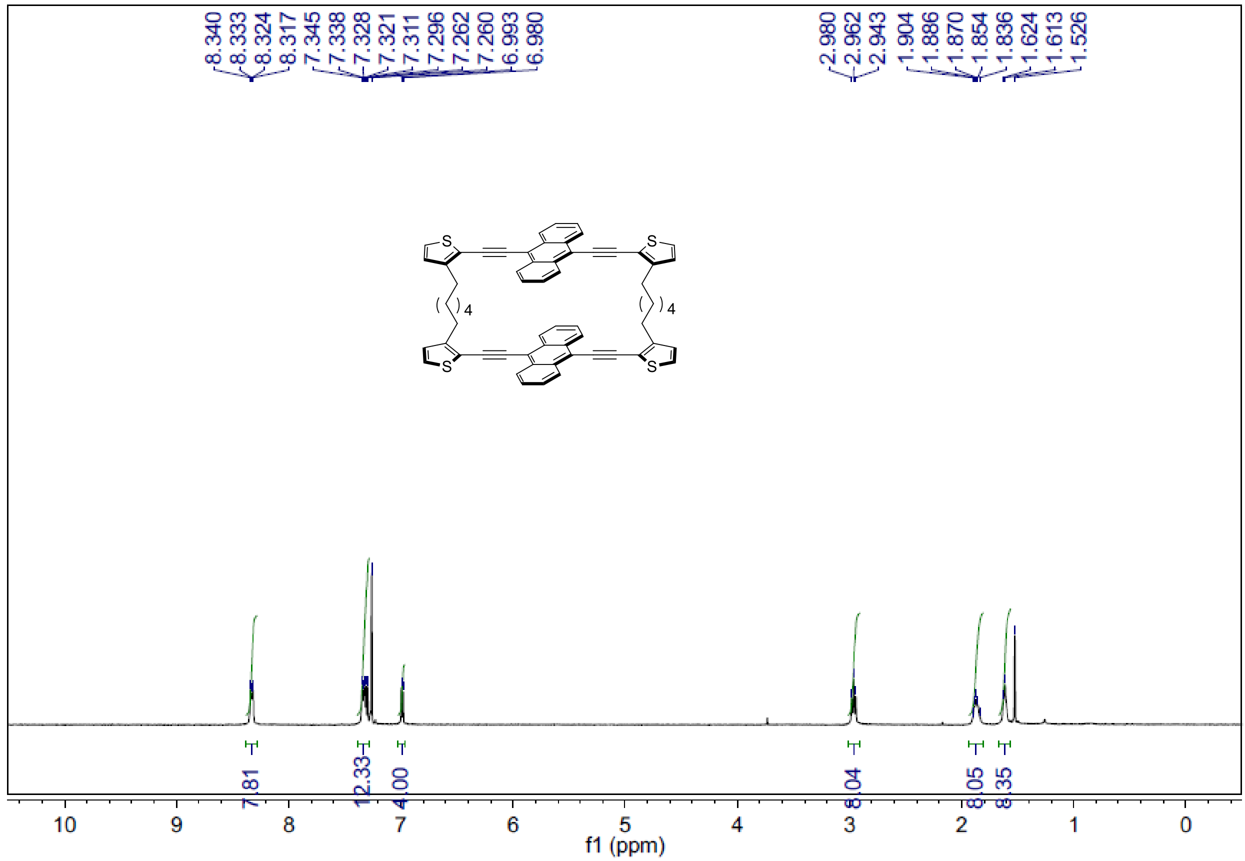

Fig. S26. ^{1}H NMR spectrum of **1a** in $CDCl_3$.

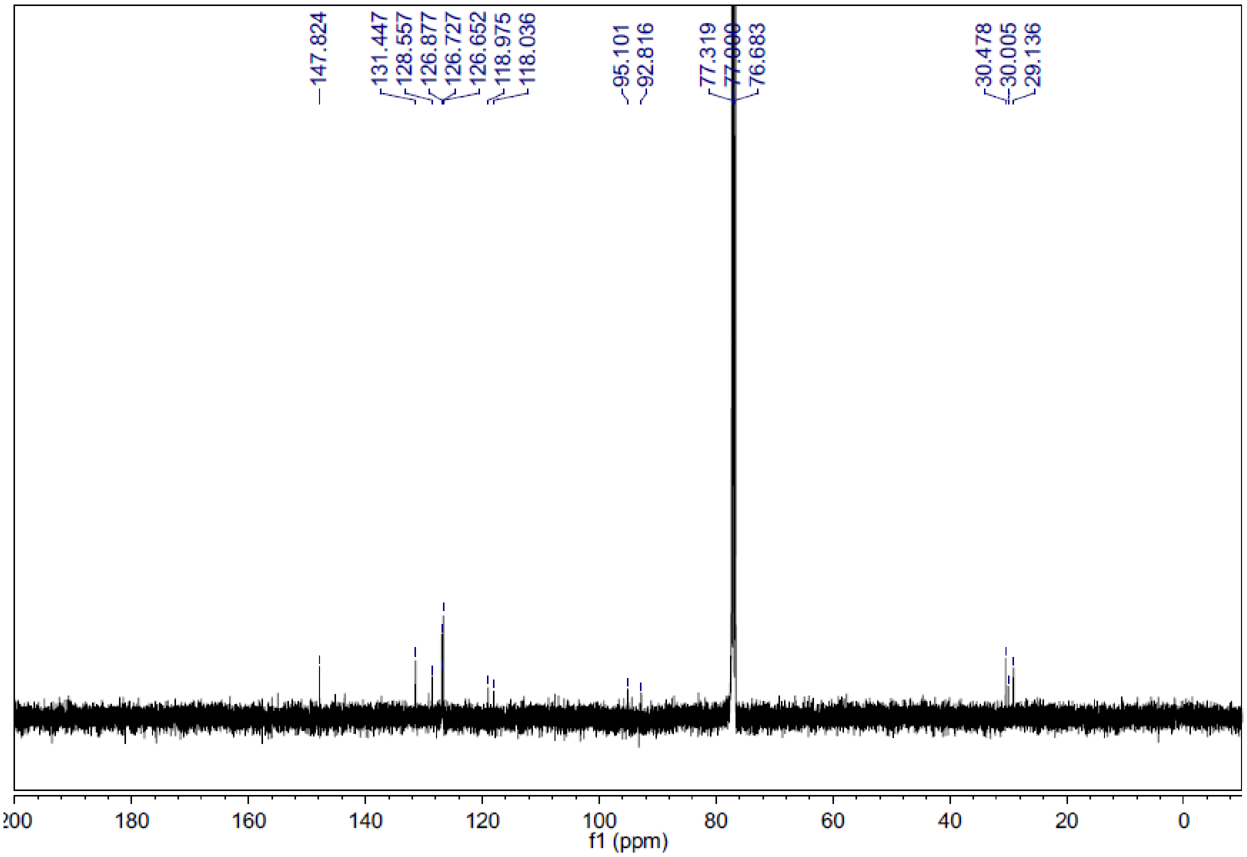

Fig. S27. ^{13}C NMR spectrum of **1a** in $CDCl_3$ (low intensity due to poor solubility).

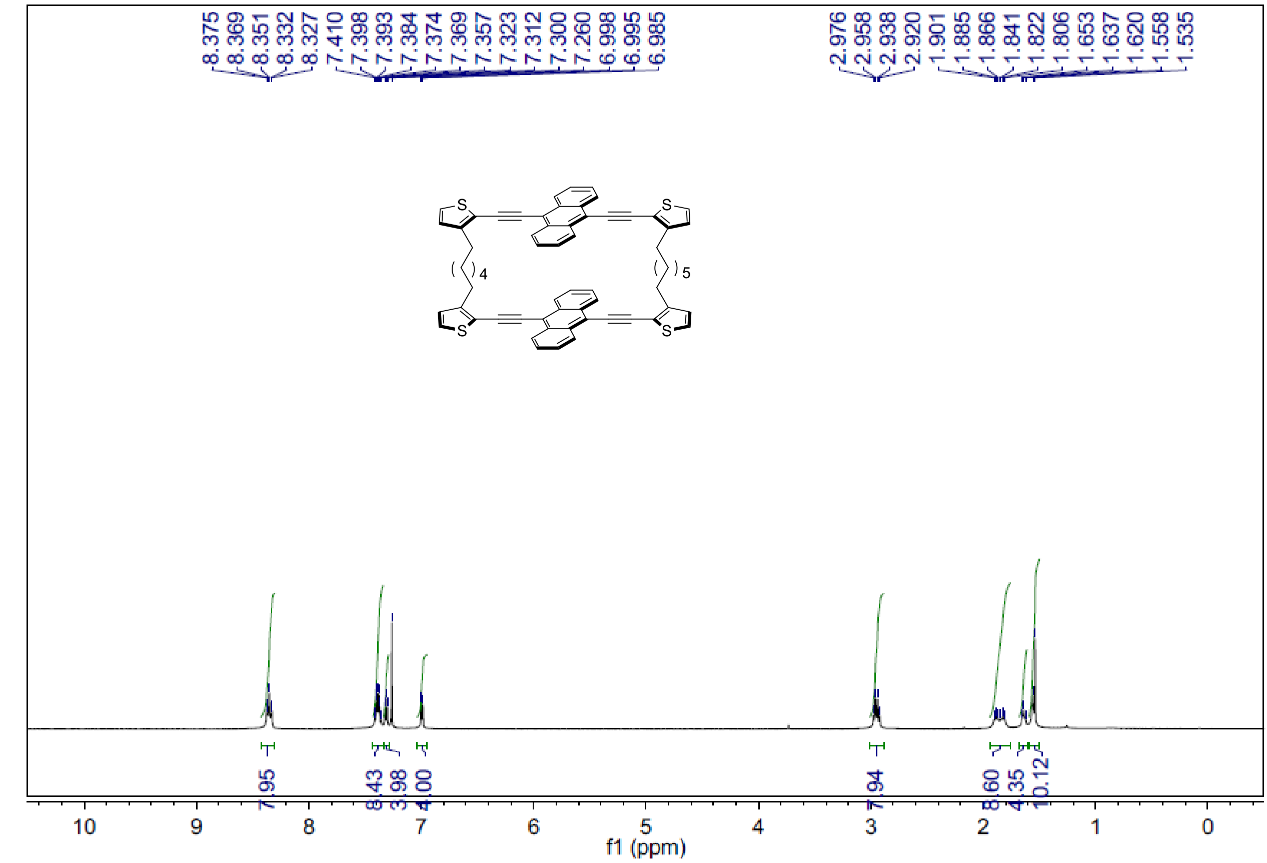

Fig. S28. ^{1}H NMR spectrum of **1b** in $CDCl_3$.

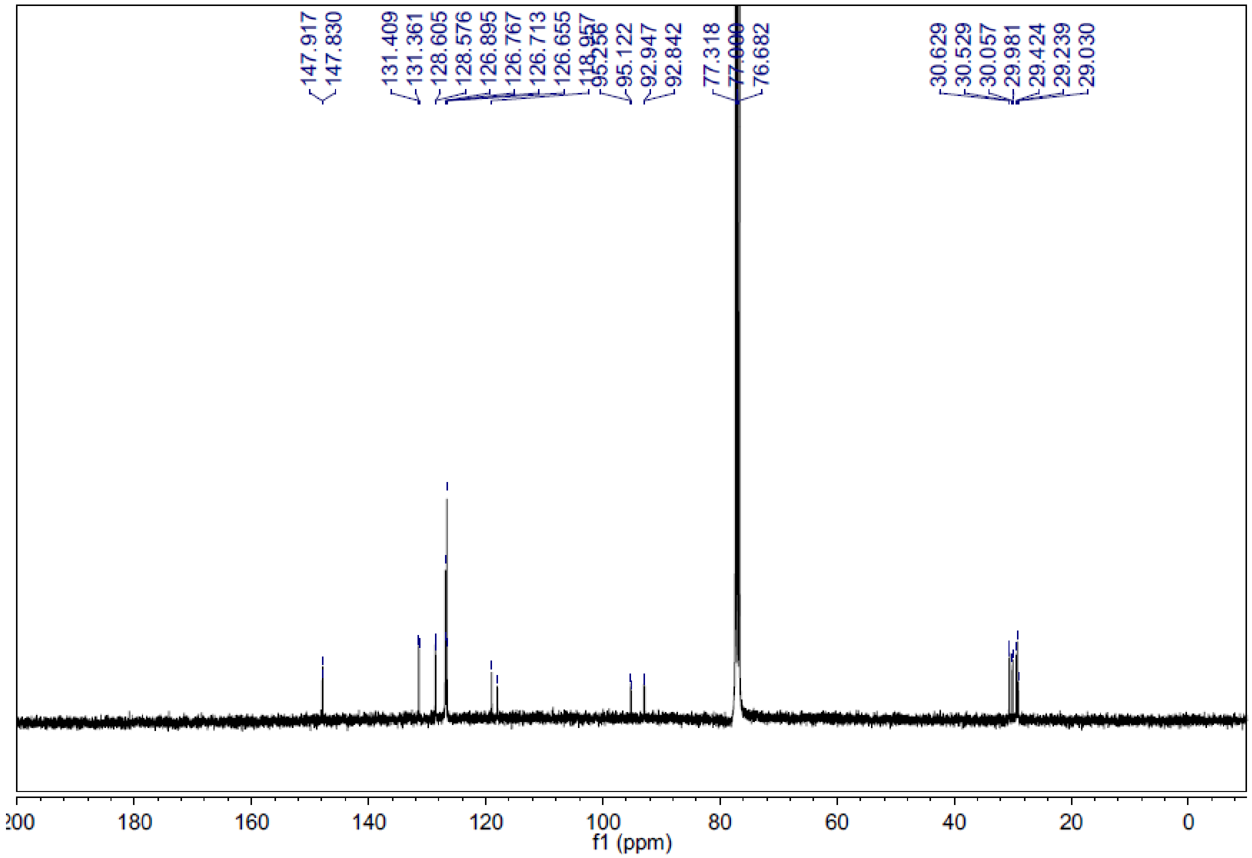

Fig. S29. ^{13}C NMR spectrum of **1b** in $CDCl_3$.

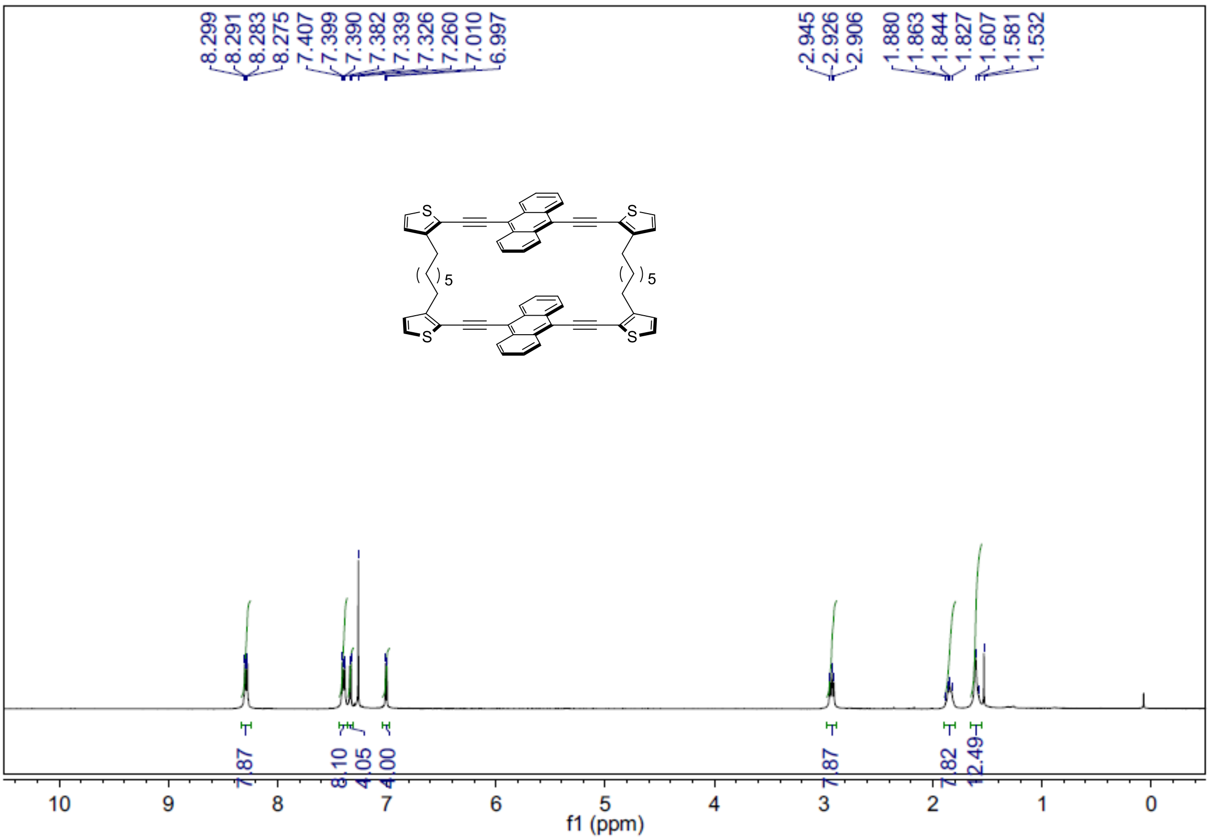

Fig. S30. ^{1}H NMR spectrum of **1c** in $CDCl_3$.

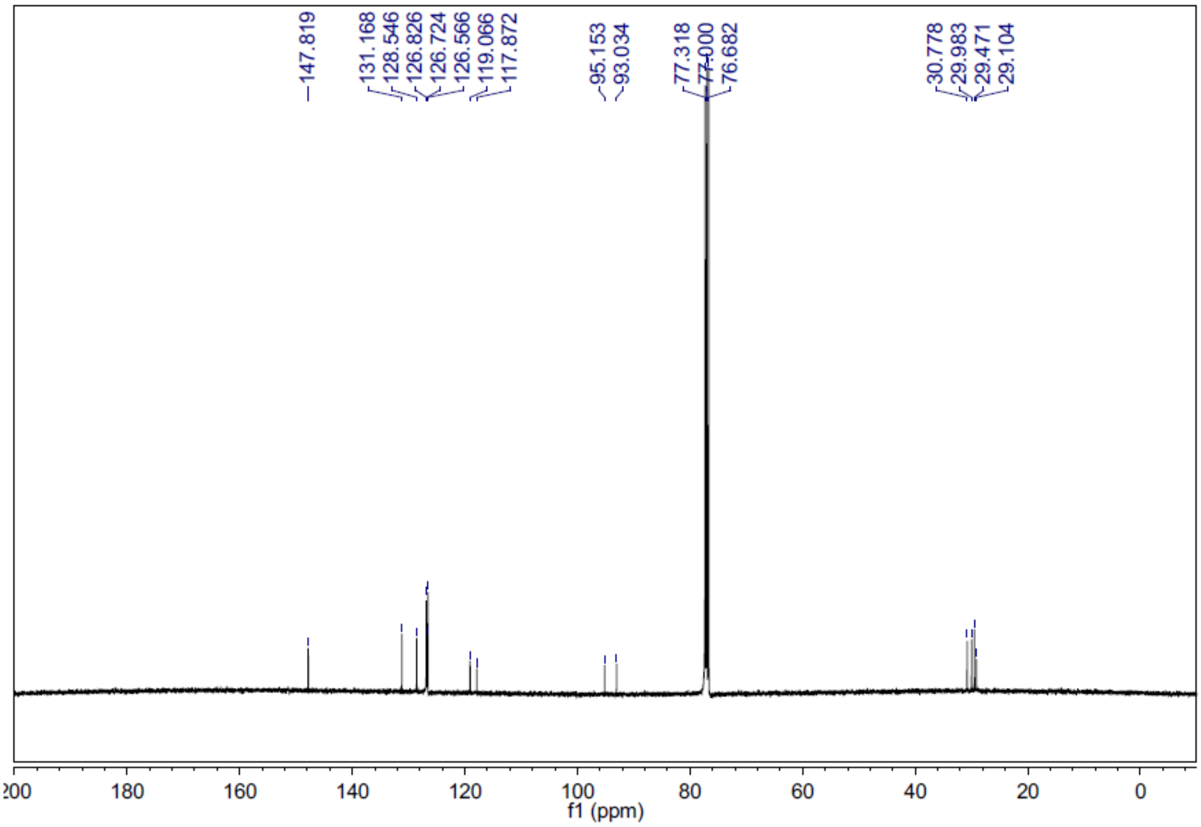

Fig. S31. ^{13}C NMR spectrum of **1c** in $CDCl_3$.
